# Integrating Tactile Feedback Technologies Into Home-Based Telerehabilitation: Opportunities and Challenges in Light of COVID-19 Pandemic

**DOI:** 10.3389/fnbot.2021.617636

**Published:** 2021-02-17

**Authors:** Shirley Handelzalts, Giulia Ballardini, Chen Avraham, Mattia Pagano, Maura Casadio, Ilana Nisky

**Affiliations:** ^1^Department of Physical Therapy, Ben-Gurion University of the Negev, Be'er Sheva, Israel; ^2^The Translational Neurorehabilitation Lab at Adi Negev Nahalat Eran, Ofakim, Israel; ^3^Department of Informatics, Bioengineering, Robotics and Systems Engineering, University of Genoa, Genoa, Italy; ^4^S.C.I.L Joint Lab, Department of Informatics, Bioengineering, Robotics and System Engineering (DIBRIS), Santa Corona Hospital, Pietra Ligure, Italy; ^5^Department of Biomedical Engineering, Ben-Gurion University of the Negev, Be'er Sheva, Israel; ^6^Zlotowski Center for Neuroscience, Ben-Gurion University of the Negev, Be'er Sheva, Israel

**Keywords:** haptic, training, stroke, neurorehabiliation, somatosensory, assessment

## Abstract

The COVID-19 pandemic has highlighted the need for advancing the development and implementation of novel means for home-based telerehabilitation in order to enable remote assessment and training for individuals with disabling conditions in need of therapy. While somatosensory input is essential for motor function, to date, most telerehabilitation therapies and technologies focus on assessing and training motor impairments, while the somatosensorial aspect is largely neglected. The integration of tactile devices into home-based rehabilitation practice has the potential to enhance the recovery of sensorimotor impairments and to promote functional gains through practice in an enriched environment with augmented tactile feedback and haptic interactions. In the current review, we outline the clinical approaches for stimulating somatosensation in home-based telerehabilitation and review the existing technologies for conveying mechanical tactile feedback (i.e., vibration, stretch, pressure, and mid-air stimulations). We focus on tactile feedback technologies that can be integrated into home-based practice due to their relatively low cost, compact size, and lightweight. The advantages and opportunities, as well as the long-term challenges and gaps with regards to implementing these technologies into home-based telerehabilitation, are discussed.

## Introduction

The COVID-19 pandemic highlights the need to accelerate the development and implementation of innovative approaches for home-based rehabilitation (Simpson and Robinson, 2020). While in normal, non-pandemic times many individuals in need of rehabilitation services do not receive sufficient therapy due to difficulties posed by the need to travel to the location where the therapy is provided, a shortage of regional rehabilitation care, and poor adherence to assignments (Cramer et al., 2019), the COVID-19 pandemic is presenting new challenges to rehabilitation services. The restrictions imposed to contain the spread of infection further limit access to rehabilitation services (Chaler et al., 2020) and challenge societal well-being. This may lead to long-term negative consequences by increasing functional impairments, and reducing participation and quality of life (Boldrini et al., 2020b). Telerehabilitation from home may partially mitigate these challenges, but state of the art telerehabilitation systems often only use visual or/and auditory feedback and lack somatosensory feedback (Navarro et al., 2018).

Somatosensory input is essential for accurate motor control and interactions with the external world (Pearson, 2000; Perez et al., 2003; Borich et al., 2015). The somatosensory impairment that is observed in many neurological disorders such as stroke, traumatic brain injury, and spinal cord injury can lead to impairments in adjusting the amount of force applied during grasping and fine manipulation of objects (Sullivan and Hedman, 2008; Doyle et al., 2010, 2014; Connell et al., 2014; Hill et al., 2014) and in performing tasks that require rapid dextrous movements (Goebl and Palmer, 2008), as well as in controlling more gross functions such as gait and posture (Maki and McIlroy, 1997; Horak, 2006).

In in-person rehabilitation intervention therapists frequently use touch to assist, and to provide and perceive information, as well as to comfort and encourage patients (Roger et al., 2002). In a survey regarding satisfaction with telerehabilitation during the COVID-19 pandemic, the absence of touch was reported by patients as a limitation (Tenforde et al., 2020). The current review focuses on tactile technologies that can be used as innovative solutions to support home-based telerehabilitation and addresses some challenges that have become more salient during the COVID-19 pandemic.

Previous reviews discussed telerehabilitation and wearable haptic devices; however, none has provided a comprehensive perspective on the variety of tactile stimulation technologies and the ways to exploit them for home-based telerehabilitation. An overview on tactile displays was conducted by Jones and Sarter (2008); however, since then significant developments in tactile technology have been presented. Culbertson et al. (2018b) reviewed the design, control, and general applications of haptic devices, but did not focus on rehabilitation applications. Several reviews focused on wearable technologies (not necessarily haptics) that can be used for remote monitoring of physiological and kinematic measurements, with a brief overview on the applications for home-based rehabilitation (Patel et al., 2012; Wang et al., 2017). Navarro et al. (2018) proposed features related to adaptive, multisensorial, physiological and social aspects that should be considered in the development process of the next generation of telerehabilitation systems. A systematic review of virtual reality technologies for rehabilitation examined the effect of haptic feedback on motor performance (Rose et al., 2018). Another review (Shull and Damian, 2015) examined wearable haptic applications for a variety of sensory impairments; however, the focus of that review was on stimulations to enhance motor performance. A previous narrative review focused on tactile technologies for hand rehabilitation in central nervous system disorders (Demain et al., 2013). In this work, we extend previous reviews by covering the development in tactile technologies over the last decade with an emphasis on wearable devices that potentially could be utilized at home. We also expand the scope to include the assessment of somatosensory deficits, in addition to various rehabilitative applications, and address the recent developments in mediation of social interaction. Specifically, we review: (1) clinical approaches for stimulating somatosensation in home-based rehabilitation, (2) tactile technologies that can be integrated into home-based rehabilitation, and (3) the challenges and gaps, as well as the opportunities, in this field.

## Clinical Approaches for Stimulating Somatosensation in Home-Based Neurorehabilitation

### Providing Tactile Augmented Feedback to Enhance Motor Control Performance and Learning

Somatosensory augmented feedback provides additional sensory cues that complement and/or replace native sensory input from the somatosensory, visual, and/or vestibular systems (Bach-y-Rita and Kercel, 2003). Tactile cues can guide patients on how to improve their movements (Bark et al., 2015) and may assist them in achieving their goals more quickly and/or more easily (Magill, 2004). A promising application of tactile feedback is to provide patients with guidance on how to improve their movements without the constant presence of a therapist (Bark et al., 2015; Bao et al., 2018), including when practicing on their own. The augmented feedback can be triggered by the participant's motor performance and can provide information continuously during the action or at specified times (Ferris and Sarter, 2011; Galambos, 2012; Kaul and Rohs, 2017). Compared with visual feedback, real time tactile feedback makes it possible for patients to receive information regarding movement errors without the need to shift visual attention, thus affording a more “natural” movement (Bark et al., 2015).

Tactile stimulation can also be beneficial even if it does not provide any information. For instance, subthreshold tactile stimulations (i.e., below the level at which a person can perceive the stimulation) add noise to proprioceptive signals and might help these signals to overcome the threshold of specific neural circuits. This phenomenon, also known as the stochastic resonance theory (Gammaitoni, 1995; Gammaitoni et al., 1998; Moss et al., 2004), facilitates more efficient detection of somatosensory information, and improves sensorimotor performance (Collins et al., 1996, 2003). As such, it could be used in the rehabilitation of individuals with sensorimotor deficits to improve motor functions (e.g., grasp, object manipulation, balance and gait) and tactile sensation (Enders et al., 2013; Seo et al., 2014, 2019).

### Applying Tactile Stimulations to Improve/Restore Cutaneous Somatosensation

Somatosensory impairment is considered to have a negative prognostic impact on rehabilitation interventions and overall motor function recovery (Bowerman et al., 2012; Dietz and Fouad, 2014; Zandvliet et al., 2020). Although the current literature in this field is limited, a recent systematic review and meta-analysis indicated positive effects in improving somatosensory impairments (Serrada et al., 2019). Specifically, sensory discrimination training by repeated practice to distinguish textures and localize tactile stimuli can influence the sensory system and drive recovery (Carey et al., 1993; Yekutiel and Guttman, 1993; Turville et al., 2019).

### Presenting Tactile Feedback in Virtual Reality Environments

Telerehabilitation is often based on virtual reality systems and interactive video games that aim to facilitate repetitions of movements and to make the repetitive exercises more engaging, enjoyable and motivating (Standen et al., 2015). The virtual experience can be further enhanced by using tactile devices that can convey haptic interactions between the user and the virtual objects (Galambos, 2012; Culbertson et al., 2018b).

### Conveying Social Tactile Interaction

Haptic feedback plays a critical role in emotional and social communication (Strong and Gaver, 1996; Brave and Dahley, 1997). During in-person rehabilitation sessions therapists often use touch to comfort and encourage patients (Roger et al., 2002). Recent developments in wearable tactile devices demonstrate very promising results in conveying sensations such as comfort and affection (Culbertson et al., 2018a; Nunez et al., 2019, 2020), attention (Baumann et al., 2010), playfulness (Mullenbach et al., 2014), or social presence (Baldi et al., 2020). The integration of social tactile aspects into telerehabilitation systems would open new possibilities for remote therapist-patient communication and may facilitate wider adoption of telerehabilitation from home by patients.

### Assessing Tactile Impairments

In addition to the above training strategies, the use of measures to quantify somatosensory deficits could help therapists to understand patients' impairments beyond motor and functional status and assist in targeting appropriate interventions. The assessment of somatosensory functions, including proprioception and sensitivity to light touch, pressure, and temperature, cannot be done remotely in the traditional way where the therapist applies the stimulation and evaluates the performance using scales. Portable, and often wearable devices that apply multimodal stimulations have the potential to provide reliable and quantitative information regarding somatosensory impairments in a home-based setting (Rinderknecht et al., 2015, 2019). Such portable devices have already been used in some virtual reality systems for baseline measurements of activity and kinematics and for tracking changes over time (Patel et al., 2012; Chen et al., 2015; Bortone et al., 2018).

## Tactile Stimulation Technologies

Over the last few decades, technologies that can provide versatile tactile stimulations have become very popular and many new devices continue to be developed. These devices can be integrated into wearable technologies and utilized for telerehabilitation due to their low cost, compact size, and lightweight. From the technological point of view, there is a variety of ways to apply tactile stimulation. These can be categorized according to the mechanism evoking the tactile sensation: mechanical, electrotactile, and thermal. In order to provide an in depth review of the technology and its applications, in this review we focus on mechanical tactile stimulations. However, it should be noted that electrotactile stimulation is also used for various assistive technologies and rehabilitation applications such as for people with visual (Bliss et al., 1970; Kajimoto et al., 2001) and auditory impairments (Weisenberger et al., 1989), as well as in prostheses, orthoses (Schweisfurth et al., 2016; Svensson et al., 2017) and stroke rehabilitation (for a review see Laufer and Elboim-Gabyzon, 2011).

Mechanical tactile stimulations can be further divided into vibration, skin deformation, and mid-air stimulations. Recently the idea of wearable tactile devices that combine vibration, stretch, and pressure for conveying multimodal haptic information was introduced (Aggravi et al., 2018; Sullivan et al., 2019; Dunkelberger et al., 2020), highlighting the importance of understanding the unique properties of each stimulation type and harnessing the advantages of each to design devices that are more than the sum of their parts. In the remainder of this section, we review the state of the art in mechanical tactile stimulation devices. For each type of device we review the technology, its applications for healthy and patient populations, and its advantages and disadvantages. The different devices and studies are summarized in Tables 1, 2. Table 1 summarizes the devices by stimulation type, actuator type, technological maturity level, and application. We rank the technological maturity level based on how extensively testing of the device has been reported in the literature, with the following levels: prototype demonstration (*N* < 10); healthy user studies (*N* = 10–100); extensive healthy user studies (*N* > 100); patient user studies (*N* = 10–100); extensive patient user studies (*N* > 100). Table 2 summarizes the studies that were reviewed here that were tested on patient populations for different rehabilitation applications.

**Table 1:** Tactile stimulation devices by type, maturity level, and applications in healthy individuals.

**Stimulation type**	**Device type**	**Device maturity level**	**Applications**	**Commercial availability**
Vibration	Single actuator	Extensive healthy and patient user studies	Improve walking pattern (Janssen et al., 2009)	BalanceFreedom of SwayStar system https://www.b2i.info/web/index.htm
			Improve force control accuracy (Ahmaniemi, 2012)	
			Convey proprioceptive information (Bark et al., 2008)	
	Multiple actuators	Extensive healthy and patient user studies	Enhance motor learning and performance (Lieberman and Breazeal, 2007; Bark et al., 2015; Kaul and Rohs, 2017; Van Breda et al., 2017; Shah et al., 2018)	
			Guide movement direction (Van Erp et al., 2005; Krueger et al., 2017; Risi et al., 2019)	
			Improve standing balance (Lee et al., 2012; Ma and Lee, 2017; Ballardini et al., 2020)	Vertiguard RT https://zeisberg.net/posturographie.html
			Improve walking pattern (Chen B. et al., 2016; Wan et al., 2016; Muijzer-Witteveen et al., 2017; Xu et al., 2017)	
			Convey various types of information (Ferris and Sarter, 2011; Cobus et al., 2018)	
			Convey affective touch (Israr and Abnousi, 2018)	
			Assess somatosensory impairments (Tommerdahl et al., 2019)	Brain Gauge https://www.corticalmetrics.com/howitworks
	Multiple actuators on a glove	Healthy and patient user studies	Convey virtual objects information (Muramatsu et al., 2012)	CyberTouch http://www.cyberglovesystems.com/cybertouch2
			Convey force information (Galambos, 2012)	
			Assess somatosensory impairments (Rinderknecht et al., 2015, 2019)	
	Single actuator with multiple probes	Healthy user studies	Assess somatosensory impairments (Holden et al., 2012; Puts et al., 2013; Mikkelsen et al., 2020)	
Skin deformation—tangential and stretch	Tactor	Extensive healthy user studies and patient studies	Alter mechanical properties of virtual objects (Sylvester and Provancher, 2007; Quek et al., 2013, 2014b; Schorr et al., 2013; Farajian et al., 2020a,b)	
			Convey direction information (Bark et al., 2010; Guinan et al., 2012, 2013a,b; Norman et al., 2014; Chinello et al., 2018; Kanjanapas et al., 2019)	
			Convey information about curvature (Frisoli et al., 2008; Prattichizzo et al., 2013), weight (Kato et al., 2016; Choi et al., 2017), and virtual objects information (Yem and Kajimoto, 2017; Wang et al., 2020)	
			Improve object manipulation (Leonardis et al., 2017; Schorr and Okamura, 2017b; Bortone et al., 2018), tracking (Quek et al., 2014b), insertion (Quek et al., 2015b), palpation (Schorr et al., 2015) and grasping (Westebring van der Putten et al., 2010; Kim and Colgate, 2012; Quek et al., 2015a; Choi et al., 2017; Stephens-Fripp et al., 2018; Avraham and Nisky, 2020; Bitton et al., 2020; Farajian et al., 2020b)	
			Guide movement direction (Bark et al., 2010; Guinan et al., 2012, 2013a,b; Norman et al., 2014; Chinello et al., 2018)	
			Improve standing balance (Hur et al., 2019)	
			Convey affective touch (Nunez et al., 2019)	
			Assess somatosensory impairments (Ballardini et al., 2018)	
	Adhesive rings	Healthy user studies	Convey affective touch (Haynes et al., 2019)	
	Belt/Vest	Healthy user studies	Substitute and augment force and torque feedback (Pacchierotti et al., 2016), convey sensation of mass (Minamizawa et al., 2007), and sensation of virtual objects (Minamizawa et al., 2008),	
			Convey direction information (Bianchi, 2016)	
			Provide feedback about grasping force (Casini et al., 2015)	
			Guide movement direction (Stanley and Kuchenbecker, 2012; Pezent et al., 2019; Smith et al., 2020) and convey path information (Kumar et al., 2017)	
			General tactile stimulation (Nakamura and Jones, 2003; Wu et al., 2010)	
	Rocker and roller	Healthy user studies	Enhance virtual object manipulation (Provancher et al., 2005)	
			Convey proprioceptive information (Battaglia et al., 2017 and 2019; Colella et al., 2019) (Clark et al., 2018)	
	Mechanical cranks	Healthy user studies	General tactile stimulation (Stephens-Fripp et al., 2018)	
Skin deformation—pressure	Indentator	Healthy and patient user studies	General tactile stimulations (Chinello et al., 2015)	
			Convey sensations of softness (Frediani and Carpi, 2020), and holding a virtual object (Merrett et al., 2011)	
			Convey direction information (Raitor et al., 2017; Agharese et al., 2018)	
			Render shape information of remote and virtual objects (Chinello et al., 2019)	
			Convey affective touch (Culbertson et al., 2018a)	
			Assess somatosensory impairments (Jacobs et al., 2000)	
	Belt	Prototype demonstration	Convey affective touch (Prattichizzo et al., 2010)	
	Pin array	Healthy and patient user studies	Create 2D and 3D graphic display (Shimizu et al., 1993; Leo et al., 2016; Brayda et al., 2018)	
			General tactile stimulations (Caldwell et al., 1999),	
			Convey sensations of roughness (Kim et al., 2009), and texture (Sarakoglou et al., 2005; Kyung and Park, 2007; Garcia-Hernandez et al., 2011)	
Skin deformation or vibration ultrasound	Mid-air technology using phased arrays	Extensive healthy user studies	Create 3D haptic shapes (Long et al., 2014; Vo and Brewster, 2015; Makino et al., 2016)	UltraLeap https://www.ultraleap.com/
			Convey affective touch (Shakeri et al., 2017, 2018)	

*Maturity level is ranked by the following levels: prototype demonstration (N < 10); healthy user studies (N = 10–100); extensive healthy user studies (N > 100); patient user studies (N = 10–100); extensive patient user studies (N > 100). Note that the applications column refers to studies in healthy individuals; for types of devices that were also tested on patients please refer to Table 2 for more detailed information about the specific studies. Commercial availability of devices that were reviewed in the current paper and tested either on healthy or patient populations*.

**Table 2:** Tactile device applications for rehabilitation.

**Application**	**Population**	**Tested in a home setting (Yes/No)**	**Type of stimulation**	**Type of device**	**Wearable/ Non-wearable**	**References**
Enhance upper extremity function	Multiple Sclerosis (*N* = 24)	No	Vibration	Multiple actuators	Wearable	Jiang et al., 2009
	Stroke (*N* = 12)	No	Subthreshold vibration	Single actuator, (TheraBracelet)	Wearable	Seo et al., 2019
Enhance gait and balance control	Stroke (*N* = 8)	No	Vibration	Multiple actuators	Wearable	Afzal et al., 2019
	Stroke (*N* = 17)	No	Vibration	Multiple actuators	Wearable	Yasuda et al., 2017
	Stroke (*N* = 3)	No	Vibration	Platform (The Rutgers Ankle Haptic Interface)	Non-wearable	Boian et al., 2003
	Stroke (*N* = 20)	No	Vibration	Multiple actuators	Wearable	Jaffe et al., 2004
	Parkinson's disease (*N* = 43)	No	Vibration	Single actuator (VibroGait)	Wearable	Fino and Mancini, 2020
	Parkinson's disease (*N* = 20)	No	Vibration	Multiple actuators (BalanceFreedom)	Wearable	Nanhoe-Mahabier et al., 2012
	Parkinson's disease (*N* = 16)	No	Pressure	Steel stick	Non-wearable	Barbic et al., 2014
	Parkinson's disease (*N* = 10)	No	Vibration	Multiple actuators (Vertiguard)	Wearable	Rossi-Izquierdo et al., 2013
	Parkinson's disease (*N* = 9) and older adults at high risk for falls (*N* = 8) and older adults (*N* = 10)	No	Vibration	Multiple actuators	Wearable	High et al., 2018
	Parkinson's disease (*N* = 9) and older adults (*N* = 9)	No	Vibration	Multiple actuators	Wearable	Lee et al., 2018
	Older adults (*N*= 12)	Yes	Vibration	Multiple actuators	Wearable	Bao et al., 2018
	Peripheral Neuropathy (*N* = 4)	No	Pressure	Ballon arrays	Wearable	McKinney et al., 2014
	Vestibular disorder (*N* = 6)	No	Vibration	Multiple actuators	Wearable	Sienko et al., 2012
	Vestibular disorder (*N* = 7)	No	Vibration	Multiple actuators	Wearable	Sienko et al., 2013
	Vestibular disorder (*N* = 13)	No	Vibration	Multiple actuators (Vertiguard)	Wearable	Brugnera et al., 2015
	Vestibular disorder (*N* = 8)	No	Vibration	Multiple actuators	Wearable	Bao et al., 2019
	Vestibular disorder (*N* = 105)	No	Vibration	Multiple actuators, (Vertiguard)	Wearable	Basta et al., 2011
Enhance tactile sensation	Stroke (*N* = 5), diabetic neuropathy (*N* = 8) and older adults (*N* = 12)	No	Subthreshold vibration	Single actuator	Non-wearable	Liu et al., 2002
	Stroke (*N* = 10)	No	Subthreshold vibration	Single actuator	Wearable	Enders et al., 2013
	Stroke (*N* = 16)	Yes	Vibration	Multiple actuators on a glove	Wearable	Seim et al., 2020b
	Digital nerve injuries (*N* = 49)	No	Pressure	Rotating disk and a card	Non-wearable	Cheng, 2000
	Chronic pain (*N* = 13)	No	Pressure	Probe	Non-wearable	Moseley et al., 2008
	Spinal cord injury (*N* = 7)	Yes	Vibration	Multiple actuators on a glove (Mobile Music Touch)	Wearable	Estes et al., 2015
Somatosensory assessment	Stroke (*N*= 2)	No	Vibration	Multiple actuators on a glove (ReHaptic Glove)	Wearable	Rinderknecht et al., 2019
	Stroke (*N* = 3)	No	Skin stretch	Tactor	Non-wearable	Ballardini et al., 2018
	Brain injury (*N* = 1)	No	Vibration	Multiple actuators (Brain Gauge)	Non-wearable	King et al., 2018
Enhance interaction realism in virtual reality environment	Children with neuromotor impairments (*N* = 20)	No	Skin stretch and pressure	Tactor	Wearable	Bortone et al., 2018
	Spinal cord injury (*N* = 9)	No	Vibration	Multiple actuators on a glove (CyberTouch)	Wearable	Dimbwadyo-Terrer et al., 2016

### Vibration

Vibration is the simplest and most common tactile stimulation technology that has become ubiquitous and is used in a wide variety of devices such as phones, watches, games, and home appliances (Culbertson et al., 2018b). Typically, the actuators used in wearable devices produce vibration at frequencies above 100 Hz, which activates the Pacinian corpuscles mechanoreceptors (Culbertson et al., 2018b). The most common locations for applying the vibrotactile stimulation are the arm (Bark et al., 2008; Huisman et al., 2013; Krueger et al., 2017; Shah et al., 2018; Risi et al., 2019) and the torso (Van Erp et al., 2005; Lee et al., 2012; Ballardini et al., 2020). Other locations for stimulation include the hand (Jiang et al., 2009; Wan et al., 2016) and different locations on the lower limb (Chen B. et al., 2016; Shi et al., 2019). The design of the device and the stimulation patterns (e.g., frequency and amplitude of the vibration) need to take into account the targeted dermatomes and the density and size of the mechanoreceptors' receptive fields which vary across the body (Jones and Sarter, 2008; Johansson and Flanagan, 2009; Shah et al., 2019) and across the skin type (e.g., hairy skin has a reduced number of Pacinian corpuscles compared to glabrous skin) (Colgate and Brown, 1994; Ackerley et al., 2014). Skin type can also influence the quality of stimulation via its mechanical properties and its physical propagation of the vibration (Dandu et al., 2019; Hachisu and Suzuki, 2019).

#### Technology

Vibrotactile feedback can be conveyed by a single actuator, or by an array of actuators that create an oscillating movement. The choice of the actuator affects the size, shape, cost, availability, robustness, speed of response, input requirements, and power consumption of the device (Choi and Kuchenbecker, 2013). An overview of the different actuators can be found in Choi and Kuchenbecker (2013) and Kern (2009).

The stimulation patterns can be divided into two fundamental categories: (1) binary on-off state, and (2) continuous vibration, created by changing parameters of the vibration signals such as amplitude, frequency, duration, rhythm, and waveform (Brewster and Brown, 2004; Jones and Sarter, 2008). Binary feedback is not continuously provided but is triggered by specific events such as an alarm or event-cue related information (Ferris and Sarter, 2011; Galambos, 2012; Kaul and Rohs, 2017). The vibration intensity can be constant or may vary according to the event (Cobus et al., 2018). Continuous vibrotactile stimulation is used to convey various types of information to the users, including: (1) state feedback, encoding position and/or velocity of limbs (Ferris and Sarter, 2011; Krueger et al., 2017; Shah et al., 2018; Risi et al., 2019), (2) force feedback, encoding the amount of force exerted (Ahmaniemi, 2012), and (3) error feedback, encoding information regarding the goal of the task and the state of the end-effector (Wall et al., 2001; Cuppone et al., 2016; Krueger et al., 2017).

By controlling the shape and timing of the signals from multiple static actuators, it is also possible to display illusions of movement that can enrich the design space of tactile stimulation. Prominent examples are: (1) phi (or beta) movement, where a smooth apparent motion of a single stimulus is created by the periodic activation of two spatially separated stimuli (Sherrick and Rogers, 1966; Lederman and Klatzky, 2009), (2) saltatory (or rabbit) illusion, i.e., illusory sweeping movement of discrete taps that occur by activating actuators in sequence (Geldard and Sherrick, 1972; Lederman and Klatzky, 2009), and (3) the tendons vibration illusion, which is an illusory perception of movement that can be evoked by triggering the muscle spindle afferents through vibrations applied to the tendon (Goodwin et al., 1972; Taylor et al., 2017).

#### Applications for Enhancing Sensorimotor Performance and Learning

In healthy individuals, vibrotactile feedback is used to enhance motor control and learning (Lieberman and Breazeal, 2007; Van Breda et al., 2017; Shah et al., 2018). It has been demonstrated that state feedback regarding the force exerted improved the accuracy of force repetition (Ahmaniemi, 2012). Other studies used state and/or error feedback to guide upper limb reaching movements in the absence of visual information (Krueger et al., 2017; Shah et al., 2018; Risi et al., 2019) and to reach accuracy levels beyond the limits of natural proprioception (Risi et al., 2019). Results from a meta-analysis indicated that vibrotactile feedback was effective in reducing task completion times, but neither forces nor errors were significantly reduced (Nitsch and Färber, 2012). In addition, vibration feedback encoding center of mass or center of pressure motion was used to improve standing balance (Lee et al., 2012; Ma and Lee, 2017; Ballardini et al., 2020) and walking patterns (Janssen et al., 2009; Muijzer-Witteveen et al., 2017; Xu et al., 2017). Vibrotactile feedback based on stochastic resonance was applied for improving visuomotor temporal integration in hand control (Nobusako et al., 2019) and balance control (Magalhães and Kohn, 2011). Vibrations that informed the users about collisions with virtual objects in a virtual reality context added realism and improved performance (Galambos, 2012; Kaul and Rohs, 2017). Also, a vibrotactile glove interface has been used to convey sensations of virtual objects (Muramatsu et al., 2012).

#### Applications in Rehabilitation

In persons with multiple sclerosis, vibrotactile feedback applied to the fingernails of the contralateral hand improved the performance of a grasping and lifting task of the more impaired hand (Jiang et al., 2009). In addition, real time state vibrotactile cues reduced postural sway during standing balance tasks and improved gait parameters after stroke (Yasuda et al., 2017; Afzal et al., 2019), in people with Parkinson's disease (Nanhoe-Mahabier et al., 2012; High et al., 2018; Lee et al., 2018; Fino and Mancini, 2020) and with vestibular disorders (Sienko et al., 2012, 2013). However, in all of these studies improvements were observed during trials, and long-term effects were not tested. Vibrotactile stimulations were also used to enhance interaction realism in a rehabilitation system based on virtual reality (Boian et al., 2003; Dimbwadyo-Terrer et al., 2016), and to avoid collisions during walking in stroke survivors (Jaffe et al., 2004).

Several randomized controlled trials (RCTs) with small cohorts tested the effect of balance training programs with vibrotactile stimulations. Following a 2-week training program using vibrotactile feedback, individuals with Parkinson's disease improved their balance control parameters and performance-based measures and retained improvements 3 months after training (Rossi-Izquierdo et al., 2013). Additionally, adults with vestibular disorders improved their balance performance and felt more confident regarding their balance while performing daily activities after a training protocol with vibrotactile stimulations compared with a control group that trained without stimulations (Brugnera et al., 2015; Bao et al., 2019). Furthermore, balance improvements were retained at 6-month follow-up assessments (Bao et al., 2019). Also, reduced body sway and improved clinical outcome measures [e.g., Sensory Organization Test (SOT) (Franchignoni et al., 2010) and Dizziness Handicap Inventory (Jacobson and Newman, 1990)] were observed in a study with a large cohort of participants with vestibular disorders (*n* = 105) who trained with vibrotactile stimulations over 2-weeks (i.e., ten-sessions) compared with a control group that trained with a sham device (Basta et al., 2011).

Subthreshold vibrotactile stimulation improved somatosensation and motor function in persons with sensorimotor impairments: stimulations applied at the wrist and dorsal hand improved the immediate fingertips light-touch sensation and grip ability of the paretic hand in stroke survivors (Enders et al., 2013). In addition, the vibrotactile detection threshold (i.e., the minimum level of vibration amplitude to be detected) at the tip of the middle finger in persons with diabetic neuropathy and stroke survivors was decreased (Liu et al., 2002). In persons with diabetic neuropathy the threshold was decreased at the foot as well (Liu et al., 2002; Khaodhiar et al., 2003). In a pilot randomized controlled trial, subthreshold vibratory stimulation was applied to the paretic wrist of stroke survivors during upper extremity task training (a total of 6 sessions provided in 2 weeks). The treatment group showed a significant improvement in hand motor function at the end of therapy, which was sustained 19 days after therapy, whereas the control group that practiced without stimulation did not improve from baseline performance (Seo et al., 2019).

**Figure 1:**
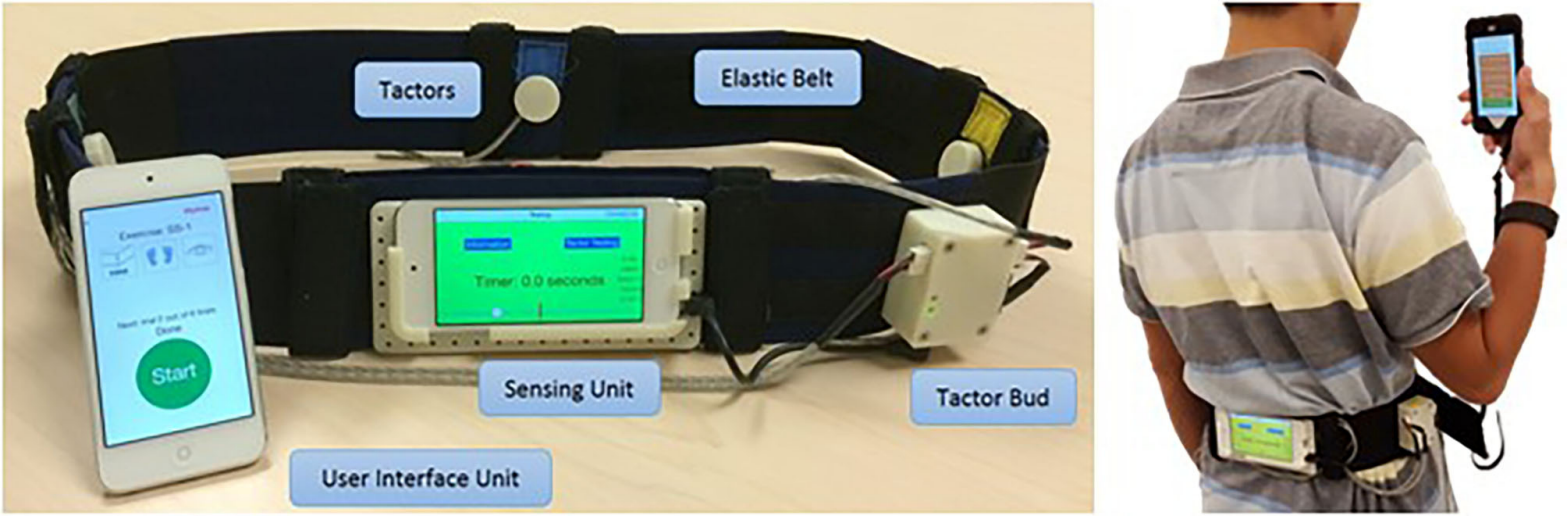
Vibrotactile stimulation devices. The smartphone-based balance trainer used in Bao et al. (2018). The sensing unit is attached to an elastic belt worn around the torso to measure trunk sway. The four tactors are aligned over the anterior, posterior, and right and left sides of the torso to provide directional vibrotactile cues.

While these studies were conducted in laboratory settings, few studies provided participants with vibrotactile devices to practice at home. Bao et al. (2018) tested the effect of long-term home-based balance training with vibrotactile sensory augmentation among community-dwelling healthy older adults. Participants were trained in static and dynamic standing and gait exercises for 8 weeks (3 sessions per week, 45-min each) using smartphone balance trainers that provided guidance while monitoring trunk sway. The experimental group received directional vibrotactile cues via actuators that were aligned around the torso in case the activation signal exceeded a pre-set threshold, while the control group practiced without supplemental feedback. Participants in the experimental group demonstrated significantly higher improvements in their SOT (Franchignoni et al., 2010) and Mini Balance Evaluation Systems Test scores (Clendaniel, 2000) compared with the control group at post training assessment. Seim et al. (2020a) designed a glove that provides subthreshold vibrotactile stimulation for stroke survivors to use at home and demonstrated the feasibility of wearing the glove for 3 h daily for 8 weeks. Also, in a double-blind RCT, chronic stroke survivors with impaired tactile sensation in the hand were given a glove to take home and asked to wear it during their normal daily routine (i.e., 3 h daily for 8 weeks) (Seim et al., 2020b). One group received a glove which provided vibrotactile stimulation to the hand and another group received a glove with the vibration disabled. Participants receiving tactile stimulations demonstrated significant improvement in tactile perception (assessed with monofilaments) in the affected hand. In another study, improvement in hand sensation was observed in participants with spinal cord injury after training with a glove providing vibration stimulations compared with participants who trained without stimulations (Estes et al., 2015). Vibration stimulations were applied during active practice sessions of playing piano in in-lab sessions (3 times a week for 30 min a session for 8 weeks) and during passive practice at home (2 h a day, 5 times a week). An illustration of a vibrotactile stimulation device is presented in Figure 1.

#### Applications for Conveying Social Tactile Cues

Gentle stroking touches resembling those of soft calming and caressing sensations are considered highly relevant in social interactions (Huisman et al., 2016). Using artificial means to convey such touches might enhance social presence in telecommunication or in virtual settings. Israr and Abnousi (2018), developed a vibrotactile device worn on the forearm that delivers stimuli which resemble caressing and calming sensations. Participants rated low frequency stimuli (<40 Hz) as pleasant sensations that feel like massaging and noted that they would be even more realistic with context. Huisman et al. (2013) developed a virtual agent setup that incorporates an augmented reality screen and a vibrotactile sleeve worn on the user's forearm. In this setup the forearm was placed under a tablet, thus allowing the user to see his/her forearm “through” the tablet. The vibrotactile stimulation combined with the visual representation of a hand touching the user created a more realistic touching illusion.

#### Assessment of Tactile Impairments

Vibrotactile simulation can be used for assessment applications. In clinical settings the duration of vibration sensation and the perception threshold are commonly measured using a tuning fork (Perkins et al., 2001; Alanazy et al., 2018). However, results regarding its reliability are variable across studies (O'Neill et al., 2006; Lai et al., 2014; Lanting et al., 2020), and, most importantly, the assessment does not quantitatively provide the degree of dysfunction and depends on the level of clinical experience (Lanting et al., 2020).

To address limitations of clinical assessments, an automated approach to quantify topesthesia (i.e., the ability to recognize the location of a tactile stimulus) was developed (Rinderknecht et al., 2015). The system consists of two wearable gloves that can apply vibrations on the hand at 24 possible locations and a touchscreen to directly indicate with the non-tested hand the precise location of perception on the tested hand. The assessment provides a standardized, repeatable measurement as well as continuous outcome measures on ratio scales (Rinderknecht et al., 2015). It was tested on healthy individuals (Rinderknecht et al., 2015) and on stroke survivors (Rinderknecht et al., 2019).

In addition, a portable vibrotactile stimulator device was used to probe tactile function through a battery of tests assessing reaction time (“press the button when you feel the vibrotactile stimulation”), threshold detection (the weakest detectable stimulus), amplitude and frequency discrimination (discriminating between two stimuli that are simultaneously applied and discriminating between the frequency of two sequentially applied stimuli). The battery targets different mechanisms of somatosensory processing (Holden et al., 2012; Puts et al., 2013; King et al., 2018; Tommerdahl et al., 2019; Mikkelsen et al., 2020). These tests were used on healthy adults and children (Puts et al., 2013) for monitoring recovery from concussion (King et al., 2018) as well as a wide range of neurological disorders (Tommerdahl et al., 2019). There are also other specific tests that aim to independently evaluate only one of the aspects investigated by this paradigm; an overview of the tests assessing vibrotactile perception in healthy subjects can be found in Jones and Sarter (2008).

#### Advantages and Disadvantages

A major advantage of vibrotactile devices is that the actuators can be easily integrated into wearable devices because they are small, lightweight, low- power, and low-cost (Alahakone and Senanayake, 2009). On the other hand, disadvantages of vibrotactile feedback stem from the properties of the mechanoreceptors activated by vibration. First, it is difficult to accurately locate the source of the stimulations if they are placed close together, because of the propagation of the vibration (Sofia and Jones, 2013; Shah et al., 2019) and the large size of the mechanoreceptors' receptive fields (Johnson et al., 2000). Second, it is difficult to convey directional information, unless several actuators are used in a spatially and/or temporally coordinated mode (Rotella et al., 2012). Third, it has been suggested that the feedback coding of some vibrotactile devices may be less effective than of others in reducing applied forces i.e., if the vibration frequency or location varies, vibrotactile feedback may be less effective in conveying information on intensity or direction than a uniform signal that alerts the user of a required response (Nitsch and Färber, 2012). Fourth, prolonged exposure to continuous vibratory stimulation could result in an unpleasant sensation (Bark et al., 2008) and has been associated with long-term nerve and tissue damage (Takeuchi et al., 1986). Also, choosing the right type, number, and target location of the actuators for patients with possible degradation of perception due to aging or disease might be challenging (Jones and Sarter, 2008).

## Skin Deformation

### Tangential Force and Skin Stretch

Tangential skin deformation is evoked by pressure of the skin against a device, combined with a lateral movement of the entire device or a small part of it. Such deformation occurs naturally when touched by a therapist, when interacting with a real object, or when a device applies forces on a user, but it may also be elicited by technological solutions specifically designed to provide tactile stimulation (Bark et al., 2009; Quek et al., 2014b; Pan et al., 2017). The stimulation is detected by the Ruffini corpuscles which are slow adapting SA-II tactile afferents in the skin that are sensitive to tangential shear strain as well as the Meissner's corpuscles which are rapid adapting RA-I tactile afferents that are sensitive to dynamic skin deformation (Johansson and Flanagan, 2009). The detection resolution of the skin stretch at the fingertip is 0.1–0.2 mm, while the direction of the stretch can be accurately perceived with less than 1.0 mm of movement (Gould et al., 1979; Greenspan and Bolanowski, 1996).

#### Technology

There are different methods to render tangential and stretch forces, e.g., a roller (Provancher et al., 2005), a belt (Minamizawa et al., 2007), or a moving tactor (Quek et al., 2013). The most common location for applying the stimulation is the finger pad (Pasquero and Hayward, 2003; Drewing et al., 2005; Gleeson et al., 2010a; Solazzi et al., 2011; Tsetserukou et al., 2014), as it contains a very high density of mechanoreceptors (Abraira and Ginty, 2013). Other locations include the palm (Guzererler et al., 2016; Ballardini et al., 2018), the forearm (Bark et al., 2008; Kuniyasu et al., 2012; Chinello et al., 2016), the arm (Casini et al., 2015; Battaglia et al., 2017), and different locations on the lower limb (Chen D. K. Y. et al., 2016; Omori et al., 2019; Wang et al., 2020). The mechanism and actuation of the device can be tailored to the desired application (see Pacchierotti et al., 2017 for a review on wearable devices). By changing the magnitude and direction of the tactile stimulations it is possible to convey different types of information such as forces and directional guidance (Biggs and Srinivasan, 2002; Paré et al., 2002; Provancher et al., 2005; Guinan et al., 2014; Bianchi, 2016; Leonardis et al., 2017; Kanjanapas et al., 2019; Bitton et al., 2020).

#### Applications for Enhancing Sensorimotor Performance and Learning

Adding a skin stretch to force feedback has been shown to affect stiffness (Quek et al., 2013, 2014a,b; Schorr et al., 2013; Farajian et al., 2020a,b) and friction (Sylvester and Provancher, 2007; Provancher and Sylvester, 2009) perception. In addition, concurrent tangential and normal skin deformation can be used to substitute and/or augment upper extremity force and torque feedback in navigation, tracking, insertion and palpation tasks (Quek et al., 2014b, 2015b; Schorr et al., 2015; Pacchierotti et al., 2016; Clark et al., 2018), generating a high fidelity haptic feedback for the sensation of mass (Minamizawa et al., 2007; Kato et al., 2016) and virtual objects (Minamizawa et al., 2008). It can also be used to enhance perception and performance in object manipulation tasks (Leonardis et al., 2017; Schorr and Okamura, 2017b), and to deliver grasp force information (Casini et al., 2015).

Skin stretch feedback providing position information improved the movement accuracy of healthy participants who controlled the movement of a virtual arm (Bark et al., 2008). Compared with vibrotactile stimulation, skin stretch feedback provided superior results, particularly when the virtual arm was in a low-inertia configuration and at low velocity (Bark et al., 2008). Gleeson et al. demonstrated the ability of healthy participants to accurately identify the direction of tangential skin deformation at the fingertip, and highlighted the potential of using skin stretch cues to aid patients with balance control impairments (Gleeson et al., 2010b). Skin stretch stimulation was also found to be effective for improving performance in a curvature discrimination task (Frisoli et al., 2008; Prattichizzo et al., 2013).

Stretching the skin can affect not only perception, but also forces that are applied by the user for stabilization. Westebring van der Putten et al. (2010) explored the influence of skin stretch and tangential deformation feedback on grasp control and demonstrated a significant improvement in pinch force control for participants who received augmented tactile feedback. Bitton et al. (2020) showed that applying tactile stimulation of the fingertips increases grip force, even in a static force maintenance task. In addition, adding an artificial skin stretch to the finger pads in the same direction as force applied by a virtual object or a haptic device increased the applied grip force (Quek et al., 2015a; Avraham and Nisky, 2020; Farajian et al., 2020b), although this effect was not seen in Quek et al. (2015b), or in the case of skin-stretch that is in the opposite direction to the external force (Avraham and Nisky, 2020).

In addition, studies have shown the ability of participants to accurately produce motion according to haptic stimuli provided by a skin stretch device (Bark et al., 2010; Stanley and Kuchenbecker, 2012; Guinan et al., 2013b; Norman et al., 2014; Chinello et al., 2018; Pezent et al., 2019; Smith et al., 2020), including in gaming applications (Guinan et al., 2012, 2013a). Skin stretch feedback encoding the velocity of postural sway along the anterior-posterior direction enhanced standing balance with perturbed sensory systems (removed vision and unreliable vestibular systems) in healthy young adults compared with conditions without skin stretch feedback (Hur et al., 2019).

In virtual reality systems, skin stretch feedback has been applied at different body locations to simulate rich physical properties during the interaction with virtual environments and objects (Minamizawa et al., 2007, 2008; Choi et al., 2017; Yem and Kajimoto, 2017; Wang et al., 2020). For example, a leg-worn device that applies varied skin stretch profiles to induce an illusory force improved the realism and enjoyment of virtual reality applications (Wang et al., 2020).

#### Applications in Rehabilitation

To date, most applications of skin stretch stimulation were demonstrated in the context of prostheses or assistive devices. For example, a multimodal tactile stimulation device helped to improve the grip force control of an electromyographic-controlled virtual prosthetic hand that was operated by targeted reinnervation amputees (Kim and Colgate, 2012). Other examples conveyed proprioceptive (Battaglia et al., 2017, 2019; Colella et al., 2019), grasp force and position information to users of prosthetic hands (Casini et al., 2015; Stephens-Fripp et al., 2018), or path information to users of a powered-wheelchair (Kumar et al., 2017). Although it has not yet been tested directly in rehabilitation protocols for neurological populations, these technologies could potentially be used for tasks such as restoration of fine object manipulation. An example of such an application was demonstrated on children with neuromotor impairments who trained in performing upper limb movements, including reach to grasp, path tracking, and hand orientation, with a wearable haptic device rendering contact forces by deformation of the fingerpad (Bortone et al., 2018).

#### Applications for Conveying Social Tactile Cues

Recently, wearable devices that can generate pleasant tactile sensations have been developed (Haynes et al., 2019; Nunez et al., 2019). A skin slip technology was used to generate an illusory sensation of continuous lateral motion that could be used to convey social touch cues, such as comfort and affection, in which stroking motions are used (Nunez et al., 2019). The stimulation was perceived as pleasant when the speed was closer to 10 cm/s and applied on the volar side of the forearm.

#### Assessment of Tactile Impairments

The assessment of tactile directional sensitivity (i.e., the ability to identify the direction of an object's motion across the skin) is considered to be a sensitive screening test of sensory function after injuries in the central or peripheral nervous system (Wall and Noordenbos, 1977; Bender et al., 1982; Hankey and Edis, 1989; Norrsell and Olausson, 1992). However, to our knowledge, assessment properties (e.g., reliability) were not tested. Recently, a skin stretch device was developed to assess somatosensory impairments at different body areas (Ballardini et al., 2018). The system offers quantitative and reliable measures of tactile acuity (i.e., testing discrimination of the direction and amplitude of skin stretch stimuli) and was validated in healthy participants and in a small cohort of stroke survivors.

#### Advantages and Disadvantages

**Figure 2:**
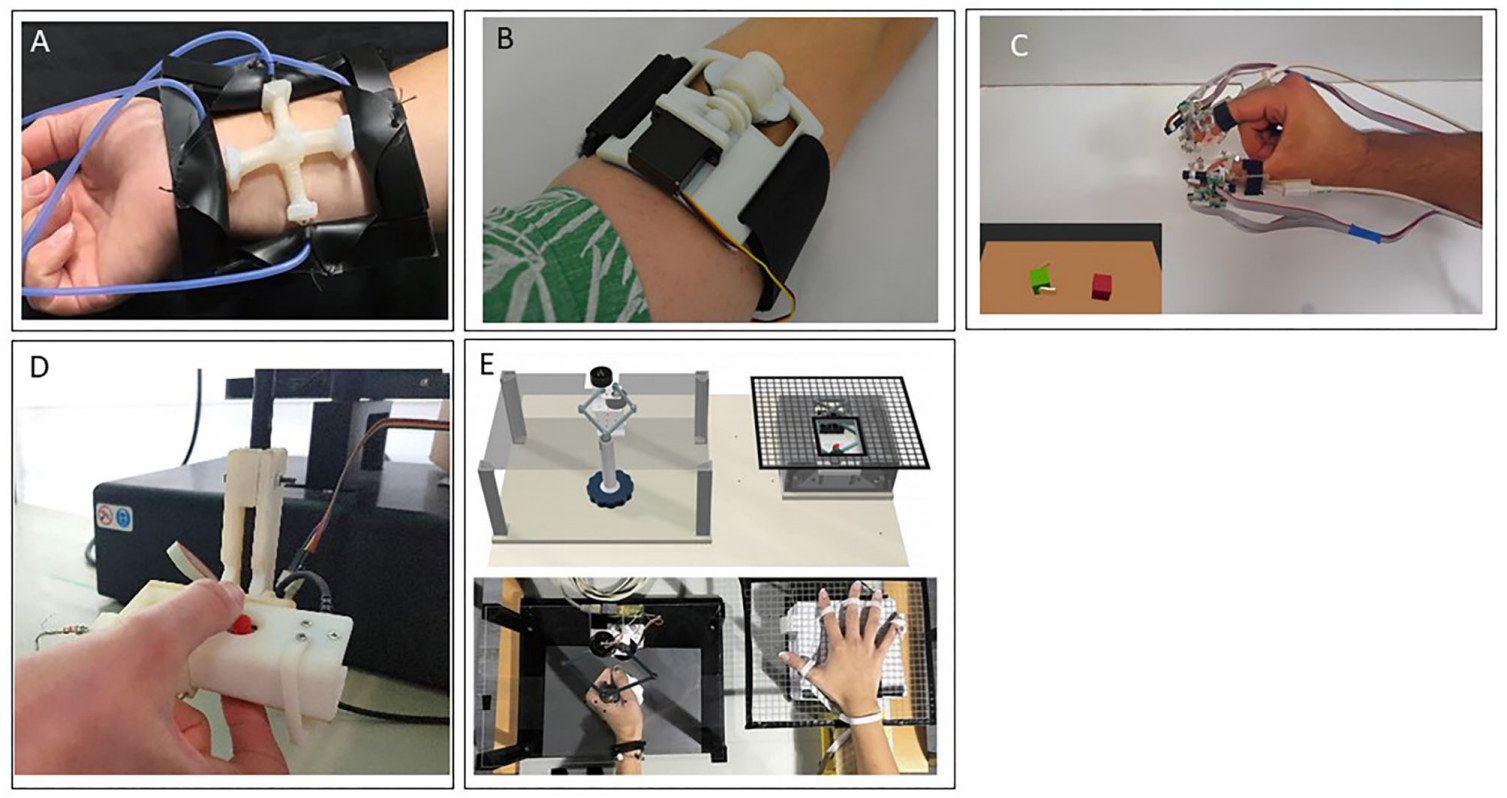
Skin deformation—tangential and stretch stimulation devices. **(A)** A wearable device that is comprised of pneumatic actuators to guide motion direction (Kanjanapas et al., 2019). **(B)** The Rice Haptic Rocker that was designed to convey proprioceptive information to users of prostheses (Battaglia et al., 2019). **(C)** A wearable device that was used to render virtual environment forces (Schorr and Okamura, 2017b). **(D)** An aperture and tactor skin-stretch device that was used to study the contribution of tactile stimulation of the fingertips to motor adaptation (Avraham and Nisky, 2020). **(E)** The Tactile-STAR, a skin brushing stimulator (top right) and a recorder (top left) that were used to assess and train tactile perception acuity. The device was specifically designed to be appropriate for use with stroke survivors who may have difficulty in maintaining contact with an aperture and tactor type of a device (Ballardini et al., 2018).

There are many advantages to skin stretch deformation. This stimulation provides a strong, quick, and accurate response to changes in skin strain (Edin, 2004). In addition, skin stretch at low frequencies is attractive for wearable devices as it does not require much power (Bark et al., 2008). It can also convey direction even with a single actuator, does not suffer from adaptation effects, and is effective even at low velocities and with small movements (Bark et al., 2008). Moreover, skin stretch feedback is effective in inducing the perception of virtual textures and illusory forces and can be used to convey intuitive proprioceptive feedback (Chossat et al., 2019). Nevertheless, skin stretch stimulation has some disadvantages. The amount of skin deformation depends on the mechanical properties of the skin and the strength of the normal forces against the actuator, and thus partial or full slippage may occur. These and other factors contribute to large inter-participant variability in the perceptual effects of skin stretch (Quek et al., 2014b; Farajian et al., 2020b), while some individuals are not at all sensitive to stretch effects (Quek et al., 2014b). Also, this type of stimulation is commonly applied at the finger pad where there is a limited area for applying the stretch. Finally, although skin stretch devices are usually safe, when developing the device, one should carefully consider unpleasant sensations and abrasion. Illustrations of tangential and stretch stimulation devices are presented in Figure 2.

### Pressure

Pressure triggers a response in the low frequency range of the slow adapting afferents SA-I, innervating the Merkel cells (Johansson and Flanagan, 2009). Technologies that provide this type of feedback deliver forces that cause deformation, and the strength of the stimulus is determined based on sensitivity thresholds, which vary across the body.

#### Technology

Pressure stimulation is commonly provided by devices that contact the skin with a single end-effector that can: (1) change its properties, such as the shape in soft actuators (Koehler et al., 2020) or the viscosity in electrorheological or magnetorheological fluids (Taylor et al., 1997; Jungmann and Schlaak, 2002; Jansen et al., 2010; Yang et al., 2010; Kim et al., 2016), (2) tighten a band around a body location, like the fingertip (Merrett et al., 2011), wrist (Stanley and Kuchenbecker, 2012) or forearm (Meli et al., 2018), and (3) press on the skin with a servomotor (Quek et al., 2015b; Schorr and Okamura, 2017a) or a hydraulic, or pneumatic actuator (Franks et al., 2008; Yem et al., 2015; Talhan and Jeon, 2018). For the latter solution, it is also possible to enlarge the area of stimulation by increasing the number of end-effectors in contact with the skin using a pin array matrix, i.e., a matrix of actuators that can be activated separately. In order to provide efficient tactile stimulation it is also important to consider the size and density of the contact points, since these will affect the cost and weight of the device, as well as its perceptual effect.

#### Applications for Enhancing Sensorimotor Performance and Learning

Using force indentation at different orientations makes it possible to display contact forces for multiple applications. Already in 1993, the technology was used to produce 2D and 3D graphic display for haptic recognition of familiar objects and was tested in blind and sighted participants (Shimizu et al., 1993; Leo et al., 2016; Brayda et al., 2018). Since then, multiple tactile devices with lightweight and compact mechanisms have been developed to produce pressure stimulation, thereby providing a range of tactile sensations including natural touch (Caldwell et al., 1999; Chinello et al., 2015; Culbertson et al., 2018a), roughness (Kim et al., 2009), softness (Frediani and Carpi, 2020), and texture (Sarakoglou et al., 2005; Kyung and Park, 2007; Kim et al., 2009; Garcia-Hernandez et al., 2011). In addition, pressure stimulation was used for conveying directional cues (Raitor et al., 2017; Agharese et al., 2018), and for rendering shape in virtual and remote environments (Chinello et al., 2019).

#### Applications in Rehabilitation

In patients with digital nerve injury, stroking, and pressing a pocket tactile stimulator and contacting the rotating disc of a tactile stimulator improved functional sensitivity measured by the smallest perceivable force using Semmes-Weinstein monofilaments (Semmes et al., 1960), and the shortest perceivable distance using a standardized two-point discrimination test instrument (Dellon et al., 1987; Cheng, 2000). In patients with complex regional pain syndrome of one limb, tactile stimulation was shown to decrease pain and increase tactile acuity when patients were required to discriminate between the type and location of tactile stimuli (Moseley et al., 2008). Skin pressure stimulation at the hallux and first metatarsal joint of the feet applied to participants with Parkinson's disease increased step length and gait velocity and reduced cadence compared with baseline measurements (Barbic et al., 2014). A wearable tactile feedback system that was originally developed for sensory augmentation of prosthetic limbs has been adapted for individuals with bilateral peripheral neuropathy (McKinney et al., 2014). Using thigh cuffs (one per leg) with silicone balloons for conveying sensory information specific to each foot, participants could modify their gait in real time (i.e., increase walking speed, step cadence and step length). Although not tested in populations undergoing rehabilitation, tactile vests worn on the torso have been shown to create a variety of tactile stimuli that could potentially be useful in applications such as balance control training (Nakamura and Jones, 2003; Wu et al., 2010). Also, pressure applied simultaneously to the thumb and index fingers generated a perception of holding an object, exhibiting the potential to provide a realistic haptic sensation in virtual reality based rehabilitation (Merrett et al., 2011).

#### Applications for Conveying Social Tactile Cues

Culbertson et al. developed a device that creates a stroking sensation using a linear array of voice coil actuators embedded in a fabric sleeve worn around the arm. The voice coils were controlled to indent the skin in a linear pattern to create the sensation of a stroking motion even though only normal force was applied (Culbertson et al., 2018a). As indicated from participants' ratings, to create a continuous and pleasant sensation the device should be controlled with a short delay and long pulse width (800 ms, 12.5% delay). Another system, the RemoTouch (Prattichizzo et al., 2010), was designed to provide experiences of remote touch. The user perceives force feedback recorded by a human that wears a glove equipped with force sensors. The measured contact force at the remote interaction is fed back to the user through wearable tactile displays for each finger. Preliminary tests show that the realism of this remote experience largely improved with the tactile feedback.

#### Assessment of Tactile Impairments

Sensitivity to pressure is often used as a measure of absolute tactile sensitivity (for more details see Demain et al., 2013). The most commonly used method to assess pressure sensation is the Semmes–Weinstein monofilaments that are calibrated to apply predetermined forces to the skin (Semmes et al., 1960; Bell-Krotoski, 1984). Jacobs et al., suggested another approach for examining the psychophysical detection threshold of pressure stimulation of a prosthetic and a normal limb (Jacobs et al., 2000). Stimulations were applied using a computer connected to a probe and to a remote control that was operated by the patient. The patient could control the amplitude of the pushing force by pressing the remote control. To measure the detection threshold the up-down method was used (i.e., the amplitude of the pushing force was decreased until the patient did not feel the stimulation and stopped pressing the remote control). Then, the amplitude was increased until 16 reversals were obtained. This setup can be modified for home-based assessment, possibly by using a smaller controller instead of the computer.

#### Advantages and Disadvantages

**Figure 3:**
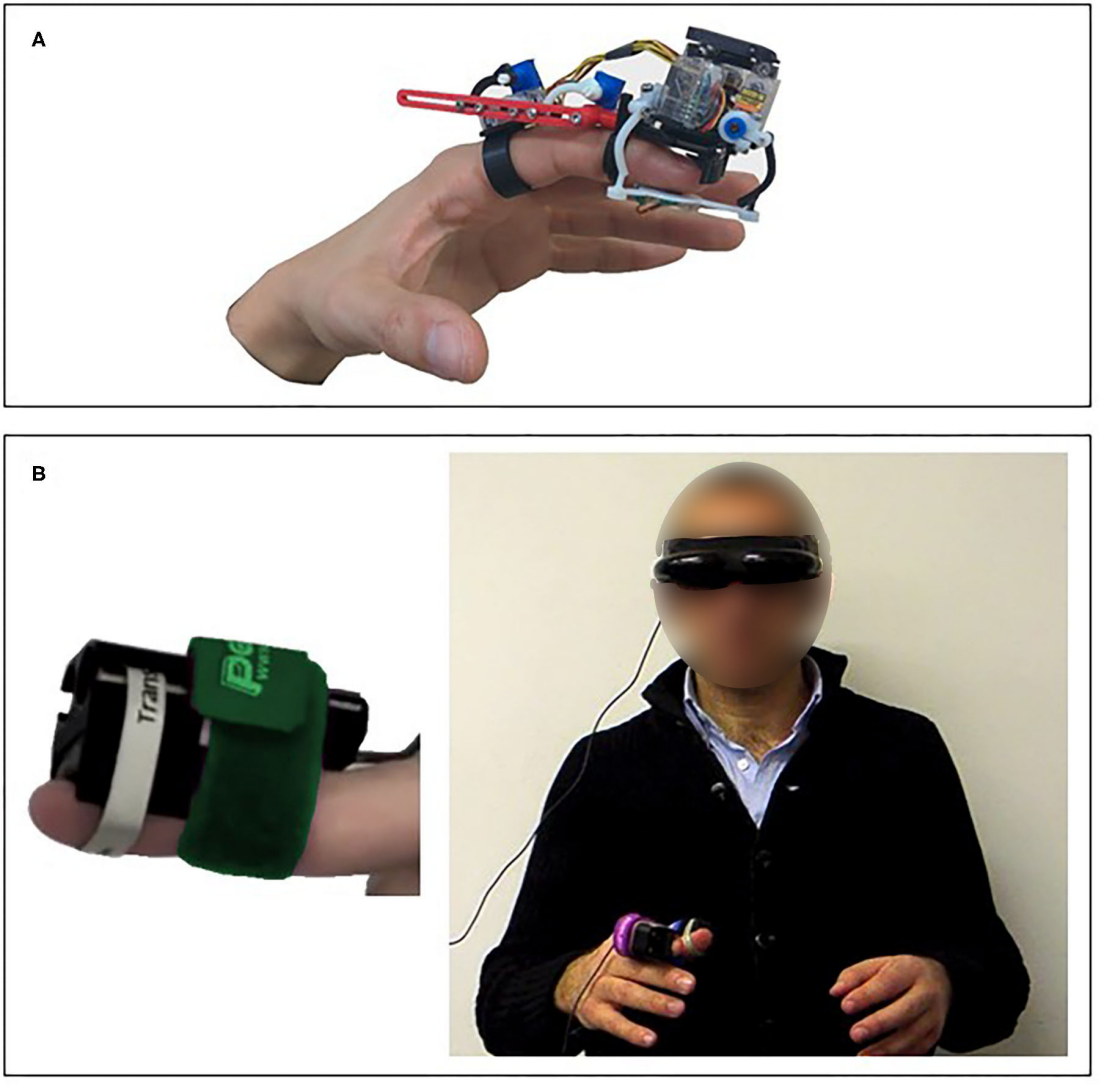
Skin deformation—pressure stimulation devices. **(A)** A wearable finger device that was designed and tested in virtual reality applications (Chinello et al., 2019). **(B)** The RemoTouch system that provides experience of remote touch (Prattichizzo et al., 2010).

Pressure stimulation enables rendering perceptual properties such as shape, curvature, orientation, and texture (Gabardi et al., 2016). However, sensitivity to pressure is largely dependent on the area of stimulation (Stevens, 1982). In addition, while multiple actuation approaches are available for applying pressure to the skin, each approach is suitable for a different application. Therefore, one should carefully consider the specifications of the design that would be appropriate for the desired application. Illustrations of pressure stimulation devices are presented in Figure 3.

### Mid-air

All the technologies described above require physical contact between the device and the body to provide somatosensory feedback, and the energy produced by the actuators is transferred to the skin through a solid medium. This allows efficient energy transduction, creating natural haptic sensations with the aid of appropriate contactors to the skin. However, these solutions present some limitations: (1) they do not exploit arbitrary body locations, i.e., can deliver feedback only at a location close to the device's end effector, (2) they may cause undesired effects due to the continuous contact between the skin and the devices, and (3) if used by different individuals, they require cleaning and disinfecting, especially in light of the recent COVID-19 related recommendations (Thomas et al., 2020). Several recent developments address these limitations by proposing mid-air technologies. They transmit the energy of the stimulus through air, avoiding the direct contact with the skin.

#### Technology

One of the main approaches to creating mid-air stimulation relies on ultrasonic waves, typically at 40 or 70 kHz frequencies (for survey see Rakkolainen et al., 2019). In this type of mid-air tactile stimulation the sensation is caused by a non-linear effect of focused ultrasound called acoustic radiation force, which induces a shear wave in the skin, creating a displacement, which triggers the mechanoreceptors within the skin and evoking mainly a pressure sensation (Gavrilov and Tsirulnikov, 2002). Most ultrasound haptic systems targeting the hand trigger the Lamellar corpuscles (Rakkolainen et al., 2019). In other body locations ultrasound can trigger other mechanoreceptors, such as Meissner corpuscles on the face (Gil et al., 2018), and Ruffini corpuscles or Merkel disks on the upper limb (Suzuki et al., 2018).

The most widely used technological solution to evoke tactile sensation with ultrasound is based on phased arrays of transducers, i.e., multiple transducers whose phase and intensity can be controlled individually, with a defined timing. In this way, the focused ultrasound waves can generate one or more localized regions of pressure in the 3D space, called focal points, without moving or turning the device. These focal points cannot be fully singular because of secondary peaks and wavelength limitations (Rakkolainen et al., 2019). However, several focal points can be controlled together to create shapes (Long et al., 2014) or textures (Monnai et al., 2015; Freeman et al., 2017). If the radiation force is modulated at the 1–1 kHz range the ultrasound waves can also evoke a vibratory sensation in addition to the pressure sensation (Hasegawa and Shinoda, 2013; Howard et al., 2020; Rutten et al., 2020).

#### Applications for Enhancing Sensorimotor Performance and Learning

The use of focused ultrasound as a non-invasive method of stimulation has been studied since the early 1970s (Gavrilov et al., 1977). Recently, this technology was used for several proof-of-concept applications, including creating floating 2D icons (Gavrilov, 2008) and 3D haptic shapes (Long et al., 2014; Monnai et al., 2014; Vo and Brewster, 2015; Makino et al., 2016), interacting in a virtual reality environment (Romanus et al., 2019; Howard et al., 2020), and gesture interaction (Shakeri et al., 2017, 2018). To the best of our knowledge, mid-air haptic devices have not yet been used for rehabilitative purposes or for somatosensory assessment.

#### Applications for Conveying Social Tactile Cues

The communication of emotions through a haptic system that uses tactile stimulation in mid-air communication was explored by Obrist et al. and showed promising results of interpretability of emotions (Obrist et al., 2015). Despite these promising results the application of ultrasound devices for conveying emotions and social interaction has not yet been extensively investigated.

#### Advantages and Disadvantages

**Figure 4:**
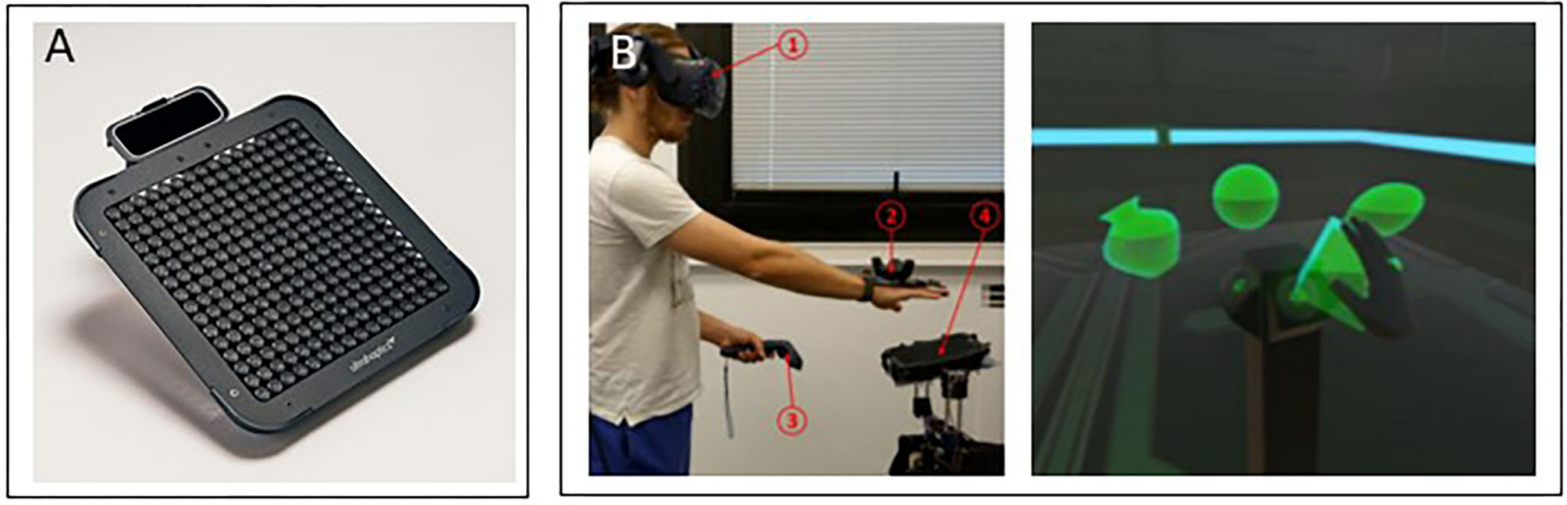
Mid-air devices. **(A)** The Ultraleap device (https://www.ultraleap.com/). **(B)** Its application to interaction with a virtual reality environment (Howard et al., 2020).

The major advantage of this emerging technology is its not requiring contact with the body, while easily and efficiently creating static or dynamic textures and volumetric shapes. Another important advantage is that commercial devices are available that use this technology, even at this early stage. In its current state, this technology has some inherent limitations that may have an impact on potential applications, including the size and the weight of the transducers (Rakkolainen et al., 2019) and the low intensity of the force conveyed to the user, which is at most 160 mN (Tsalamlal et al., 2013), and so does not allow the rendering of real-word interaction forces. Nevertheless, we anticipate that mid-air solutions will develop in the next few years, and we foresee that they will be designed for rehabilitation purposes and clinical assessments. Illustrations of mid-air stimulation devices are presented in Figure 4.

## Discussion

The COVID-19 pandemic is currently placing significant pressure on health services including rehabilitation services, worldwide. The reduced access to rehabilitation care due to restrictions as well as the reduction in rehabilitation services as a consequence of reassignment of rehabilitation professionals to acute care and the transformation of rehabilitation facilities into makeshift inpatient wards (Boldrini et al., 2020a; Chaler et al., 2020) are expected to lead to long-lasting negative consequences for individuals with disabilities (Boldrini et al., 2020b). In fact, these are only the tip of the iceberg when considering the long-standing and more severe problem of limited resources in hospital care together with the rising number of individuals with chronic diseases (Koh et al., 2015; Steihaug et al., 2016; Dodakian et al., 2017).

Remote communication technologies, as well as technologies developed for home-based telerehabilitation, have the potential to support neurorehabilitation care and make breakthroughs in treatment by facilitating continuous and intensive training. The emerging technological solutions reviewed in the current paper highlight the promise of wearable tactile stimulation devices to enhance home-based rehabilitation training gains by the provision of tactile feedback and haptic interactions. These technologies seem propitious and attractive for home-based rehabilitation: the devices are wearable, portable, and relatively low cost (estimated cost between tens and hundreds of dollars). Moreover, some of these technologies can easily be integrated into virtual/telerehabilitation environments (Feintuch et al., 2006; Bortone et al., 2018; Wang et al., 2020).

However, despite technological advantages and great potential for home-based practice, to date, tactile feedback devices have not yet evolved into common solutions for rehabilitation. There are still challenges that need to be met in a joint effort between sensorimotor neuroscientists, technology developers and clinicians in order to successfully integrate tactile technologies into neurorehabilitation programs. We review these challenges in the remainder of this section.

### Testing Training Effects on Large Patient Populations

Most tactile device prototypes were tested on healthy individuals or on small cohorts of patients and their effects need to be further examined: (1) on larger patient populations, ideally in randomized controlled trials, (2) over longer training periods, and with long-term follow up assessments to evaluate whether improvements observed immediately post training have been retained after training is completed and (3) with respect to outcome measures relevant to the daily life function of the patients. Studies conducted on healthy individuals often focus on laboratory parameters, while in patients undergoing rehabilitation exploring whether training effects have transferred to daily life activities is of clinical significance. As was demonstrated above, few such examples exist in the literature; however, these are the exception and not the rule, and more studies are needed. Several factors contribute to the difficulty of overcoming this challenge. First, the lack of collaborations between technology developers, researchers, clinicians, and rehabilitation facilities. Second, it is difficult to secure funding for such large-scale studies. Third, the facts that most tactile devices are not commercially available and do not have medical device safety approval limits the ability to easily test them on patient populations.

### Translation Into Clinical Practice

To integrate tactile stimulations into rehabilitation training it is critical to identify the optimal method to provide the feedback and the patients that would benefit from such training. The feedback provided by some common devices might be difficult to interpret and integrate. Also, the tactile stimuli patterns might not be intuitive or might be too complex for the user, due to either the number of tactile motors forcing the user to process a redundant set of signals, or to the encoding methods that may require specific attention (Brewster and Brown, 2004; Ballardini et al., 2020). This is especially important for patients undergoing rehabilitation training, who are often at the initial stages of learning that already require a relatively high degree of cognitive effort and attention (Fitts and Posner, 1967). Moreover, some neurological patients suffer from cognitive and attention deficits, and hence, to benefit from added information, the feedback must be simple (Van Vliet and Wulf, 2006). Additionally, the cognitive load of interpreting tactile cues in applications where the patient's attention is divided among multiple tasks, and how this might reduce the saliency of the cues, should be further explored (Gleeson et al., 2010b; Shah et al., 2018).

The optimal timing of providing somatosensory feedback also needs to be examined. For example, providing feedback for the entire duration of training can improve short term performance, but may limit motor learning. Conversely, providing feedback for only portions of training might produce poor initial performance, but improve motor skill retention (Winstein and Schmidt, 1990). Moreover, the conditions under which tactile feedback is most effective at improving task performance should be examined (e.g., whether it is most effective when supplementing another modality), as well as the temporal and spatial patterns and the location for applying the stimulation.

In addition, affective haptic feedback, used to render realistic feelings, has the potential to enhance remote patient-therapist communication. It can also be applied to reinvigorate the patient's interest when he/she is bored or frustrated during practice (Eid and Al Osman, 2015). While wearable haptic devices were designed to replicate a specific interaction or gesture such as comfort and affection (Culbertson et al., 2018a; Nunez et al., 2019, 2020), attention (Baumann et al., 2010) or social presence (Baldi et al., 2020), further exploration is needed in order to gain a better understanding of how to create realistic sensations, how to display them in complete synchronization with other display modalities (i.e., visual, auditory, olfactory, etc.), and how to integrate them in the right context during remote rehabilitation sessions. Other important challenges relate to touch etiquette in social interaction and how to incorporate social, cultural and individual differences with respect to the acceptance and meaning of affective touch (Eid and Al Osman, 2015).

### Using the Technology at Home

Although the devices seem promising for home use and some have already been tested in at-home practice (Bao et al., 2018; Seim et al., 2020a,b) some gaps still need to be bridged in this regard. First, further studies are needed to explore the feasibility of using tactile devices by patients undergoing home-based telerehabilitation: whether patients can correctly wear and operate the device without assistance, whether the form of the device is compatible for patients with different impairments, the adherence of using or wearing the device (Seim et al., 2020a), and safety and technical problems that may arise when using it during the training period (Seo et al., 2020). Second, tactile devices need to be integrated into already existing or new telerehabilitation/virtual reality systems to provide the whole framework of sensorimotor training (Feintuch et al., 2006).

Rehabilitation platforms that are capable of intelligent, adaptable tactile feedback configurations, adjustable in terms of difficulty level, capable of measuring performance and progression and of providing exercises relevant to daily living activities as well as motivating the user's engagement could provide a more tailored training intervention to maximize improvements (Shull and Damian, 2015; Navarro et al., 2018). Additionally, there are other important issues related to telerehabilitation in general, such as web communication between the therapist and the patient, information security, and data storing that are beyond the technical-clinical outlook of our review.

## Conclusions

The COVID-19 pandemic has highlighted the need for home-based telerehabilitation and at the same time has accelerated the adoption of a digital culture worldwide. Exploiting this opportunity together with the rapid developments in wearable haptic technologies offers a time window to advance sensorimotor neurorehabilitation, elevating it to innovative solutions for home-based therapies. Although there remain gaps and challenges that still need to be addressed jointly by scientists, technology developers and clinicians, wearable haptic devices, if correctly adapted, could potentially turn into cost-effective medical devices for use at home by individuals in need of rehabilitation treatments. The integration of tactile devices into home-based telerehabilitation practice has the potential to enhance patients' functional gains and quality of life through practice in an enriched environment with augmented tactile feedback and tactile interactions.

## Author Contributions

All authors contributed to the article (drafting and writing) and approved the submitted version.

## Conflict of Interest

The authors declare that the research was conducted in the absence of any commercial or financial relationships that could be construed as a potential conflict of interest.

## References

[B1] Abraira V. E. Ginty D. D. (2013). The sensory neurons of touch. Neuron 79, 618–639. 10.1016/j.neuron.2013.07.051 23972592 PMC3811145

[B2] Ackerley R. Carlsson I. Wester H. Olausson H. Backlund Wasling H. (2014). Touch perceptions across skin sites: differences between sensitivity, direction discrimination and pleasantness. Front. Behav. Neurosci. 8:54. 10.3389/fnbeh.2014.00054 24600368 PMC3928539

[B3] Afzal M. R. Lee H. Eizad A. Lee C. H. Oh M. K. Yoon J. (2019). Effects of vibrotactile biofeedback coding schemes on gait symmetry training of individuals with stroke. IEEE Trans. Neural Syst. Rehabil. Eng. 27, 1617–1625 10.1109/TNSRE.2019.2924682 31247557

[B4] Aggravi M. Pausé F. Giordano P. R. Pacchierotti C. (2018). Design and evaluation of a wearable haptic device for skin stretch, pressure, and vibrotactile stimuli. IEEE Robot. Autom. Lett. 3, 2166–2173. 10.1109/LRA.2018.2810887

[B5] Agharese N. Cloyd T. Blumenschein L. H. Raitor M. Hawkes E. W. Culbertson H.. (2018). HapWRAP: soft growing wearable haptic device, in IEEE International Conference on Robotics and Automation (ICRA) (Brisbane, QLD), 5466–5472.

[B6] Ahmaniemi T. (2012). Effect of dynamic vibrotactile feedback on the control of isometric finger force. IEEE Trans. Haptics 6, 376–380. 10.1109/TOH.2012.72 24808334

[B7] Alahakone A. U. Senanayake S. M. N. A. (2009). Vibrotactile feedback systems: Current trends in rehabilitation, sports and information display, Paper presented at the IEEE/ASME International Conference on Advanced Intelligent Mechatronics, AIM (Singapore: IEEE), 1148–1153. 10.1109/AIM.2009.5229741

[B8] Alanazy M. H. Alfurayh N. A. Almweisheer S. N. Aljafen B. N. Muayqil T. (2018). The conventional tuning fork as a quantitative tool for vibration threshold. Muscle Nerve 57, 49–53. 10.1002/mus.25680 28466970

[B9] Avraham C. Nisky I. (2020). The effect of tactile augmentation on manipulation and grip force control during force-field adaptation. J. Neuroeng. Rehabil. 17, 1–19. 10.1186/s12984-020-0649-y 32046743 PMC7014637

[B10] Bach-y-Rita P. W. Kercel S. (2003). Sensory substitution and the human–machine interface. Trends Cogn. Sci. 7, 541–546. 10.1016/j.tics.2003.10.013 14643370

[B11] Baldi T. L. Paolocci G. Barcelli D. Prattichizzo D. (2020). Wearable haptics for remote social walking. IEEE Trans. Haptics 13, 761–776. 10.1109/TOH.2020.2967049 31944997

[B12] Ballardini G. Carlini G. Giannoni P. Scheidt R. A. Nisky I. Casadio M. (2018). Tactile-STAR: a novel tactile STimulator and recorder system for evaluating and improving tactile perception. Front. Neurorobot. 12:12. 10.3389/fnbot.2018.00012 29681809 PMC5897626

[B13] Ballardini G. Florio V. Canessa A. Carlini G. Morasso P. Casadio M. (2020). Vibrotactile feedback for improving standing balance. Front. Bioeng. Biotechnol. 8:94. 10.3389/fbioe.2020.00094 32154229 PMC7046798

[B14] Bao T. Carender W. J. Kinnaird C. Barone V. J. Peethambaran G. Whitney S. L.. (2018). Effects of long-term balance training with vibrotactile sensory augmentation among community-dwelling healthy older adults: a randomized preliminary study. J. Neuroeng. Rehabil. 15:5. 10.1186/s12984-017-0339-6 29347946 PMC5774163

[B15] Bao T. Klatt B. N. Carender W. J. Kinnaird C. Alsubaie S. Whitney S. L.. (2019). Effects of long-term vestibular rehabilitation therapy with vibrotactile sensory augmentation for people with unilateral vestibular disorders–A randomized preliminary study. J. Vestib. Res. 29, 323–334. 10.3233/VES-190683 31609716 PMC9249282

[B16] Barbic F. Galli M. Dalla Vecchia L. Canesi M. Cimolin V. Porta A.. (2014). Effects of mechanical stimulation of the feet on gait and cardiovascular autonomic control in parkinson's disease. J. Appl. Physiol. 116, 495–503. 10.1152/japplphysiol.01160.2013 24436294

[B17] Bark K. Hyman E. Tan F. Cha E. Jax S. A. Buxbaum L. J.. (2015). Effects of vibrotactile feedback on human learning of arm motions. IEEE Trans. Neural Syst. Rehabil. Eng. 23, 51–63. 10.1109/TNSRE.2014.2327229 25486644 PMC4623827

[B18] Bark K. Wheeler J. Lee G. Savall J. Cutkosky M. (2009). A wearable skin stretch device for haptic feedback, in World Haptics 2009-Third Joint EuroHaptics conference and Symposium on Haptic Interfaces for Virtual Environment and Teleoperator Systems (Salt Lake City, UT), 464–469. 10.1109/WHC.2009.4810850

[B19] Bark K. Wheeler J. Shull P. Savall J. Cutkosky M. (2010). Rotational skin stretch feedback: a wearable haptic display for motion. IEEE Trans. Haptics 3, 166–176. 10.1109/TOH.2010.21 27788071

[B20] Bark K. Wheeler J. W. Premakumar S. Cutkosky M. R. (2008). Comparison of skin stretch and vibrotactile stimulation for feedback of proprioceptive information, in Haptics Interfaces for Virtual Environment and Teleoperator Systems Symposium (Reno, NV), 71–78. 10.1109/HAPTICS.2008.4479916

[B21] Basta D. Rossi-Izquierdo M. Soto-Varela A. Greters M. E. Bittar R. S. Steinhagen-Thiessen E.. (2011). Efficacy of a vibrotactile neurofeedback training in stance and gait conditions for the treatment of balance deficits: a double-blind, placebo-controlled multicenter study. Otol. Neurotol. 32, 1492–1499. 10.1097/MAO.0b013e31823827ec 22089958

[B22] Battaglia E. Clark J. P. Bianchi M. Catalano M. G. Bicchi A. O'Malley M. K. (2017). The rice haptic rocker: skin stretch haptic feedback with the Pisa/IIT SoftHand, in 2017 IEEE World Haptics Conference (WHC), 7–12.

[B23] Battaglia E. Clark J. P. Bianchi M. Catalano M. G. Bicchi A. O'Malley M. K. (2019). Skin stretch haptic feedback to convey closure information in anthropomorphic, under-actuated upper limb soft prostheses. IEEE Trans. Haptics 12, 508–520. 10.1109/TOH.2019.2915075 31071053

[B24] Baumann M. A. MacLean K. E. Hazelton T. W. McKay A. (2010). Emulating human attention-getting practices with wearable haptics, in 2010 IEEE Haptics Symposium (Waltham, MA), 149–156.

[B25] Bell-Krotoski J. A. (1984). Light touch-deep pressure testing using Semmes-Weinstein monofilaments. in Rehabilitation of the Hand, 3rd Edn,” eds Hunter J. M. Schnieder L. H. Mackin E. J. Bell JA J. A. (St. Louis, MO: CV Mosby), 585–593.

[B26] Bender M. B. Stacy C. Cohen J. (1982). Agraphesthesia. A disorder of directional cutaneous kinesthesia or a disorientation in cutaneous space. J. Neurol. Sci. 53, 531–555. 6279783 10.1016/0022-510x(82)90249-0

[B27] Bianchi M. (2016). A fabric-based approach for wearable haptics. Electronics 5:44. 10.3390/electronics5030044

[B28] Biggs J. Srinivasan M. A. (2002). Tangential versus normal displacements of skin: Relative effectiveness for producing tactile sensations, in Proceedings 10th Symposium on Haptic Interfaces for Virtual Environment and Teleoperator Systems. HAPTICS (Orlando, FL), 121–128.

[B29] Bitton G. Nisky I. Zarrouk D. (2020). A novel grip force measurement concept for tactile stimulation mechanisms - design, validation, and user study. arXiv Preprint arXiv:2006.04053. 10.1109/TOH.2020.3037175 33180733

[B30] Bliss J. C. Katcher M. H. Rogers C. H. Shepard R. P. (1970). Optical-to-tactile image conversion for the blind. IEEE Trans. Man Mach. Syst. 11, 58–65. 6027744

[B31] Boian R. F. Deutsch J. E. Burdea G. C. Lewis J. (2003). Haptic effects for virtual reality-based post-stroke rehabilitation, in 11th Symposium on Haptic Interfaces for Virtual Environment and Teleoperator Systems, HAPTICS. Proceedings (Los Angeles, CA), 247–253.

[B32] Boldrini P. Bernetti A. Fiore P. SIMFER Executive Committee (2020a). Impact of COVID-19 outbreak on rehabilitation services and Physical and Rehabilitation Medicine physicians' activities in Italy. An official document of the Italian PRM Society (SIMFER). Eur. J. Phys. Rehabil. Med. 56, 316–318. 10.23736/S1973-9087.20.06256-5 32175719

[B33] Boldrini P. Garcea M. Brichetto G. Reale N. Tonolo S. Falabella V.. (2020b). Living with a disability during the pandemic. “instant paper from the field” on rehabilitation answers to the COVID-19 emergency. Eur. J. Phys. Rehabil. Med. 56, 331–334. 10.23736/S1973-9087.20.06373-X 32406226

[B34] Borich M. R. Brodie S. M. Gray W. A. Ionta S. Boyd L. A. (2015). Understanding the role of the primary somatosensory cortex: opportunities for rehabilitation. Neuropsychologia 79(Pt B), 246–255. 10.1016/j.neuropsychologia.2015.07.007 26164474 PMC4904790

[B35] Bortone I. Leonardis D. Mastronicola N. Crecchi A. Bonfiglio L. Procopio C.. (2018). Wearable haptics and immersive virtual reality rehabilitation training in children with neuromotor impairments. IEEE Trans. Neural Syst. Rehabil. Eng. 26, 1469–1478. 10.1109/TNSRE.2018.2846814 29985156

[B36] Bowerman C. C. Semrau J. A. Kiss Z. Dukelow S. P. (2012). The importance of somatosensory deficits in neurological disease, in International Functional Electrical Stimulation Society (IFESS) Conference (Banff, AB), 2–5.

[B37] Brave S. Dahley A. (1997). inTouch: a medium for haptic interpersonal communication, in CHI '97: CHI '97 Extended Abstracts on Human Factors in Computing Systems (New York, NY: ACM Press), 363–364.

[B38] Brayda L. Leo F. Baccelliere C. Ferrari E. Vigini C. (2018). Updated tactile feedback with a pin array matrix helps blind people to reduce self-location errors. Micromachines 9:351. 10.3390/mi9070351 30424284 PMC6082250

[B39] Brewster S. A. Brown L. M. (2004). Tactons: structured tactile messages for non-visual information display, in Proc. Fifth Australasian User Interface Conference (AUIC2004), ed Cockburn A. (Dunedin: ACS), 15–23.

[B40] Brugnera C. Bittar R. S. M. Greters M. E. Basta D. (2015). Effects of vibrotactile vestibular substitution on vestibular rehabilitation - preliminary study. Braz. J. Otorhinolaryngol. 81, 616–621. 10.1016/j.bjorl.2015.08.013 26480904 PMC9442680

[B41] Caldwell D. G. Tsagarakis N. Giesler C. (1999). An integrated tactile/shear feedback array for stimulation of finger mechanoreceptor, in Proceedings 1999 IEEE International Conference on Robotics and Automation (Cat. No. 99CH36288C) Vol. 1 (IEEE), 287–292.

[B42] Carey L. M. Matyas T. A. Oke L. E. (1993). Sensory loss in stroke patients: effective training of tactile and proprioceptive discrimination. Arch. Phys. Med. Rehabil. 74, 602–611. 8503750 10.1016/0003-9993(93)90158-7

[B43] Casini S. Morvidoni M. Bianchi M. Catalano M. Grioli G. Bicchi A. (2015). Design and realization of the CUFF - clenching upper-limb force feedback wearable device for distributed mechano-tactile stimulation of normal and tangential skin forces, in 2015 IEEE/RSJ International Conference on Intelligent Robots and Systems (IROS) (Hamburg). 10.1109/IROS.2015.7353520

[B44] Chaler J. Gil Fraguas L. Gómez García A. Laxe S. Luna Cabrera F. Llavona R.. (2020). Impact of coronavirus disease 2019 outbreak on rehabilitation services and physical rehabilitation medicine and rehabilitation physicians' activities: perspectives from the spanish experience. Eur. J. Phys. Rehabil. Med. 56, 369–371. 10.23736/S1973-9087.20.06304-2 32329590

[B45] Chen B. Feng Y. Wang Q. (2016). Combining vibrotactile feedback with volitional myoelectric control for robotic transtibial prostheses. Front. Neurorobot. 10:8. 10.3389/fnbot.2016.00008 27597824 PMC4993021

[B46] Chen D. K. Y. Anderson I. A. Walker C. G. Besier T. F. (2016). Lower extremity lateral skin stretch perception for haptic feedback. IEEE Trans. Haptics 9, 62–68. 10.1109/TOH.2016.2516012 26761902

[B47] Chen Y. Garcia-Vergara S. Howard A. M. (2015). Effect of a home-based virtual reality intervention for children with cerebral palsy using super pop VR evaluation metrics: a feasibility study. Rehabil. Res. Pract. 2015:812348. 10.1155/2015/812348 26457202 PMC4589626

[B48] Cheng A. S. (2000). Use of early tactile stimulation in rehabilitation of digital nerve injuries. Am. J. Occup. Ther. 54, 159–165. 10.5014/ajot.54.2.159 10732177

[B49] Chinello F. Malvezzi M. Pacchierotti C. Prattichizzo D. (2015). Design and development of a 3RRS wearable fingertip cutaneous device., in IEEE International Conference on Advanced Intelligent Mechatronics (AIM) (Busan), 293–298.

[B50] Chinello F. Malvezzi M. Prattichizzo D. Pacchierotti C. (2019). A modular wearable finger interface for cutaneous and kinesthetic interaction: control and evaluation. IEEE Trans. Indus. Electron. 67, 706–716. 10.1109/TIE.2019.2899551

[B51] Chinello F. Pacchierotti C. Bimbo J. Tsagarakis N. G. Prattichizzo D. (2018). Design and evaluation of a wearable skin stretch device for haptic guidance. IEEE Robot. Autom. Lett. 3, 524–531. 10.1109/LRA.2017.2766244

[B52] Chinello F. Pacchierotti C. Tsagarakis N. G. Prattichizzo D. (2016). Design of a wearable skin stretch cutaneous device for the upper limb. in IEEE Haptics Symposium (HAPTICS) (Philadelphia, PA), 14–20.

[B53] Choi I. Culbertson H. Miller M. R. Olwal A. Follmer S. (2017). Grabity: a wearable haptic interface for simulating weight and grasping in virtual reality, in Proceedings of the 30th Annual ACM Symposium on User Interface Software and Technology (Québec City, QC), 119–130.

[B54] Choi S. Kuchenbecker K. J. (2013). Vibrotactile display: perception, technology, and applications. Proc. IEEE 101, 2093–2104. 10.1109/JPROC.2012.2221071

[B55] Chossat J. B. Chen D. K. Y. Park Y. L. Shull P. B. (2019). Soft wearable skin-stretch device for haptic feedback using twisted and coiled polymer actuators. IEEE Trans. Haptics 12, 521–532. 10.1109/TOH.2019.2943154 31562105

[B56] Clark J. P. Kim S. Y. O'Malley M. K. (2018). The rice haptic rocker: comparing longitudinal and lateral upper-limb skin stretch perception, in International Conference on Human Haptic Sensing and Touch Enabled Computer Applications (Pisa), 125–134.

[B57] Clendaniel R. A. (2000). Outcome measures for assessment of treatment of the dizzy and balance disorder patient. Otolaryngol. Clin. North Am. 33, 519–533. 10.1016/s0030-6665(05)70225-5 10815035

[B58] Cobus V. Ehrhardt B. Boll S. Heuten W. (2018). Vibrotactile alarm display for critical care, in Proceedings of the 7th ACM International Symposium on Pervasive Displays (Munich), 1–7.

[B59] Colella N. Bianchi M. Grioli G. Bicchi A. Catalano M. G. (2019). A novel skin-stretch haptic device for intuitive control of robotic prostheses and avatars. IEEE Robot. Autom. Lett. 4, 1572–1579. 10.1109/LRA.2019.2896484

[B60] Colgate J. E. Brown J. M. (1994). Factors affecting the z-width of a haptic display, in Proceedings of the IEEE International Conference on Robotics and Automation (San Diego, CA), 3205–3210.

[B61] Collins J. J. Imhoff T. T. Grigg P. (1996). Noise-enhanced tactile sensation. Nature 383:770. 8893000 10.1038/383770a0

[B62] Collins J. J. Priplata A. A. Gravelle D. C. Niemi J. Harry J. Lipsitz L. A. (2003). Noise-enhanced human sensorimotor function. IEEE Eng. Med. Biol. Mag. 22, 76–83. 10.1109/memb.2003.1195700 12733463

[B63] Connell L. A. McMahon N. E. Adams N. (2014). Stroke survivors' experiences of somatosensory impairment after stroke: an interpretative phenomenological analysis. Physiotherapy 100, 150–155. 10.1016/j.physio.2013.09.003 24239191

[B64] Cramer S. C. Dodakian L. Le V. See J. Augsburger R. McKenzie A.. (2019). Efficacy of home-based telerehabilitation vs in-clinic therapy for adults after stroke: a randomized clinical trial. JAMA Neurol. 76, 1079–1087. 10.1001/jamaneurol.2019.1604 31233135 PMC6593624

[B65] Culbertson H. Nunez C. M. Israr A. Lau F. Abnousi F. Okamura A. (2018a). A social haptic device to create continuous lateral motion using sequential normal indentation, in 2018 IEEE Haptics Symposium (HAPTICS) (San Francisco, CA). 10.1109/HAPTICS.2018.8357149

[B66] Culbertson H. Schorr S. B. Okamura A. M. (2018b). Haptics: the present and future of artificial touch sensation. Annu. Rev. Control Robot. Auton. Syst. 1, 385–409. 10.1146/annurev-control-060117-105043

[B67] Cuppone A. V. Squeri V. Semprini M. Masia L. Konczak J. (2016). Robot-assisted proprioceptive training with added vibro-tactile feedback enhances somatosensory and motor performance. PLoS ONE 11:e0164511. 10.1371/journal.pone.0164511 27727321 PMC5058482

[B68] Dandu B. Shao Y. Stanley A. Visell Y. (2019). Spatiotemporal haptic effects from a single actuator via spectral control of cutaneous wave propagation, in IEEE World Haptics Conference (WHC) (Tokyo), 425-430.

[B69] Dellon A. L. Mackinnon S. E. Crosby P. M. (1987). Reliability of two-point discrimination measurements. J. Hand Surg. 12(5 Pt 1), 693–696. 10.1016/s0363-5023(87)80049-7 3655225

[B70] Demain S. Metcalf C. D. Merrett G. V. Zheng D. Cunningham S. (2013). A narrative review on haptic devices: relating the physiology and psychophysical properties of the hand to devices for rehabilitation in central nervous system disorders. Disab. Rehabil. Assist. Technol. 8, 181–189. 10.3109/17483107.2012.697532 22794937

[B71] Dietz V. Fouad K. (2014). Restoration of sensorimotor functions after spinal cord injury. Brain 137(Pt 3), 654–667. 10.1093/brain/awt262 24103913

[B72] Dimbwadyo-Terrer I. Trincado-Alonso F. de los Reyes-Guzmán A. Aznar M. A. Alcubilla C. Pérez-Nombela S.. (2016). Upper limb rehabilitation after spinal cord injury: a treatment based on a data glove and an immersive virtual reality environment. Disabil. Rehabil. Assist. Technol. 11, 462–467. 10.3109/17483107.2015.1027293 26181226

[B73] Dodakian L. McKenzie A. L. Le V. See J. Pearson-Fuhrhop K. Burke Quinlan E.. (2017). A home-based telerehabilitation program for patients with stroke. Neurorehabil. Neural Repair 31, 923–933. 10.1177/1545968317733818 29072556 PMC5734923

[B74] Doyle S. Bennett S. Fasoli S. E. McKenna K. T. (2010). Interventions for sensory impairment in the upper limb after stroke. Cochrane Database Syst. Rev. 6:CD006331. 10.1002/14651858.CD006331.pub2 20556766 PMC6464855

[B75] Doyle S. D. Bennett S. Dudgeon B. (2014). Upper limb post-stroke sensory impairments: the survivor's experience. Disabil. Rehabil. 36, 993–1000. 10.3109/09638288.2013.825649 23971679

[B76] Drewing K. Fritschi M. Zopf R. Ernst M. O. Buss M. (2005). First evaluation of a novel tactile display exerting shear force via lateral displacement. ACM Trans. Appl. Percept. 2, 118–131. 10.1145/1060581.1060586

[B77] Dunkelberger N. Sullivan J. L. Bradley J. Manickam I. Dasarathy G. Baraniuk R. G.. (2020). A multi-sensory approach to present phonemes as language through a wearable haptic device. IEEE Trans. Haptics. 10.1109/TOH.2020.3009581 32746381

[B78] Edin B. B. (2004). Quantitative analyses of dynamic strain sensitivity in human skin mechanoreceptors. J. Neurophysiol. 92, 3233–3243. 10.1152/jn.00628.2004 15548636

[B79] Eid M. A. Al Osman H. (2015). Affective haptics: current research and future directions. IEEE Access 4, 26–40. 10.1109/ACCESS.2015.2497316

[B80] Enders L. R. Hur P. Johnson M. J. Seo N. J. (2013). Remote vibrotactile noise improves light touch sensation in stroke survivors' fingertips via stochastic resonance. J. Neuroeng. Rehabil. 10:105. 10.1186/1743-0003-10-105 24112371 PMC3852405

[B81] Estes L. T. Backus D. Starner T. (2015). A wearable vibration glove for improving hand sensation in persons with spinal cord injury using passive haptic rehabilitation, in 2015 9th International Conference on Pervasive Computing Technologies for Healthcare (PervasiveHealth) (Istanbul), 37–44.

[B82] Farajian M. Kossowsky H. Leib R. Nisky I. (2020a). Visual feedback weakens the augmentation of perceived stiffness by artificial skin stretch. bioRxiv 2020.07.22.215715. 10.1101/2020.07.22.215715 33465030

[B83] Farajian M. Leib R. Kossowsky H. Zaidenberg T. Mussa-Ivaldi F. A. Nisky I. (2020b). Stretching the skin immediately enhances perceived stiffness and gradually enhances the predictive control of grip force. Elife 9:e52653. 10.7554/eLife.52653 32292163 PMC7176431

[B84] Feintuch U. Raz L. Hwang J. Josman N. Katz N. Kizony R.. (2006). Integrating haptic-tactile feedback into a video-capture-based virtual environment for rehabilitation. Cyberpsychol. Behav. 9, 129–132. 10.1089/cpb.2006.9.129 16640464

[B85] Ferris T. K. Sarter N. (2011). Continuously informing vibrotactile displays in support of attention management and multitasking in anesthesiology. Hum. Factors 53, 600–611. 10.1177/0018720811425043 22235523

[B86] Fino P. C. Mancini M. (2020). Phase-dependent effects of closed-loop tactile feedback on gait stability in parkinson's disease. IEEE Trans. Neural Syst. Rehabil. Eng. 28, 1636–1641. 10.1109/TNSRE.2020.2997283 32634100 PMC7771239

[B87] Fitts P. M. Posner M. I. (1967). Human Performance. Belmont, CA: Brooks/Cole.

[B88] Franchignoni F. Horak F. Godi M. Nardone A. Giordano A. (2010). Using psychometric techniques to improve the balance evaluation systems test: the mini-BESTest. J. Rehabil. Med. 42, 323–331. 10.2340/16501977-0537 20461334 PMC3228839

[B89] Franks J. Culjat M. King C. H. Franco M. Bisley J. Grundfest W.. (2008). Pneumatic balloon actuators for tactile feedback in robotic surgery. Indus. Robot. 35, 449–455.

[B90] Frediani G. Carpi F. (2020). Tactile display of softness on fingertip. Sci. Rep. 10:20491. 10.1038/s41598-020-77591-0 33235252 PMC7686500

[B91] Freeman E. Anderson R. Williamson J. Wilson G. Brewster S. A. (2017). Textured surfaces for ultrasound haptic displays, in Proceedings of the 19th ACM International Conference on Multimodal Interaction (Glasgow), 491–492.

[B92] Frisoli A. Solazzi M. Salsedo F. Bergamasco M. (2008). A fingertip haptic display for improving curvature discrimination. Presence Teleoperators Virtual Environ. 17, 550–561. 10.1162/pres.17.6.550

[B93] Gabardi M. Solazzi M. Leonardis D. Frisoli A. (2016). A new wearable fingertip haptic interface for the rendering of virtual shapes and surface features, in 2016 IEEE Haptics Symposium (HAPTICS) (Philadelphia, PA). 10.1109/HAPTICS.2016.7463168

[B94] Galambos P. (2012). Vibrotactile feedback for haptics and telemanipulation: survey, concept and experiment. Acta Polytech. Hung. 9, 41–65.

[B95] Gammaitoni L. (1995). Stochastic resonance and the dithering effect in threshold physical systems. Phys. Rev. E. 52:4691. 10.1103/PhysRevE.52.4691 9963964

[B96] Gammaitoni L. Hänggi P. Jung P. Marchesoni F. (1998). Stochastic resonance. Rev. Mod. Phys. 70:223. 10.4249/scholarpedia.1474

[B97] Garcia-Hernandez N. Sarakoglou I. Tsagarakis N. Caldwell D. (2011). Orientation discrimination of patterned surfaces through an actuated and non-actuated tactile display, in IEEE World Haptics Conference (Istanbul), 599–604. 10.1109/WHC.2011.5945553

[B98] Gavrilov L. R. (2008). The possibility of generating focal regions of complex configurations in application to the problems of stimulation of human receptor structures by focused ultrasound. Acoust. Phys. 54, 269–278. 10.1134/S1063771008020152

[B99] Gavrilov L. R. Gersuni G. V. Ilyinski O. B. Tsirulnikov E. M. Shchekanov E. E. (1977). A study of reception with the use of focused ultrasound. I. effects on the skin and deep receptor structures in man. Brain Res. 135, 265–277. 10.1016/0006-8993(77)91030-7 922476

[B100] Gavrilov L. R. Tsirulnikov E. M. (2002). Mechanisms of stimulation effects of focused ultrasound on neural structures: role of nonlinear effects. Nonlinear Acoust. 445–448.

[B101] Geldard F. A. Sherrick C. E. (1972). The cutaneous “rabbit”: a perceptual illusion. Science 178, 178–179. 10.1126/science.178.4057.178 5076909

[B102] Gil H. Son H. Kim J. Oakley I. (2018). Whiskers: exploring the use of ultrasonic haptic cues on the face, in Proceedings of the CHI Conference on Human Factors in Computing Systems (Montréal, QC), 1–13.

[B103] Gleeson B. T. Horschel S. K. Provancher W. R. (2010a). Design of a fingertip-mounted tactile display with tangential skin displacement feedback. IEEE Trans. Haptics 3, 297–301. 10.1109/TOH.2010.8 27780150

[B104] Gleeson B. T. Horschel S. K. Provancher W. R. (2010b). Perception of direction for applied tangential skin displacement: effects of speed, displacement, and repetition. IEEE Trans. Haptics 3, 177–188. 10.1109/TOH.2010.20 27788072

[B105] Goebl W. Palmer C. (2008). Tactile feedback and timing accuracy in piano performance. Exp. Brain Res. 186, 471–479. 10.1007/s00221-007-1252-1 18193412

[B106] Goodwin G. M. McCloskey D. I. Matthews P. B. (1972). Proprioceptive illusions induced by muscle vibration: contribution by muscle spindles to perception? Science 175, 1382–1384. 10.1126/science.175.4028.1382 4258209

[B107] Gould W. R. Vierck C. J. Luck M. M. (1979). Cues supporting recognition of the orientation or direction of movement of tactile stimuli, in Sensory Functions of the Skin of Humans (Boston, MA: Springer), 63–78.

[B108] Greenspan J. D. Bolanowski S. J. (1996). Chapter 2 - The psychophysics of tactile perception and its peripheral physiological basis, in Pain and Touch, Handbook of Perception and Cognition, ed Kruger L. (San Diego, CA: Academic Press), 25–103. 10.1016/B978-012426910-1/50004-2

[B109] Guinan A. L. Caswell N. A. Drews F. A. Provancher W. R. (2013a). A video game controller with skin stretch haptic feedback, in IEEE International Conference on Consumer Electronics (ICCE) (Las Vegas, NV), 456–457.

[B110] Guinan A. L. Hornbaker N. C. Montandon M. N. Doxon A. J. Provancher W. R. (2013b). Back-to-back skin stretch feedback for communicating five degree-of-freedom direction cues, in World Haptics Conference (WHC) (Daejeon), 13–18.

[B111] Guinan A. L. Montandon M. N. Caswell N. A. Provancher W. R. (2012). Skin stretch feedback for gaming environments, in IEEE International Workshop on Haptic Audio Visual Environments and Games (HAVE) Proceedings (Munich), 101–106.

[B112] Guinan A. L. Montandon M. N. Doxon A. J. Provancher W. R. (2014). Discrimination thresholds for communicating rotational inertia and torque using differential skin stretch feedback in virtual environments, in IEEE Haptics Symposium (HAPTICS) (Houston, TX), 277–282.

[B113] Guzererler A. Provancher W. R. Basdogan C. (2016). Perception of skin stretch applied to palm: effects of speed and displacement, in Haptics: Perception, Devices, Control, and Applications, Lecture Notes in Computer Science, eds Bello F. Kajimoto H. Visel Y. (Cham: Springer International Publishing), 180–189. 10.1007/978-3-319-42321-0_17

[B114] Hachisu T. Suzuki K. (2019). Representing interpersonal touch directions by tactile apparent motion using smart bracelets. IEEE Trans. Haptics 12, 327–338. 10.1109/TOH.2019.2929810 31352354

[B115] Hankey G. J. Edis R. H. (1989). The utility of testing tactile perception of direction of scratch as a sensitive clinical sign of posterior column dysfunction in spinal cord disorders. J. Neurol. Neurosurg. Psychiatry 52, 395–398. 2926427 10.1136/jnnp.52.3.395 PMC1032418

[B116] Hasegawa K. Shinoda H. (2013). Aerial display of vibrotactile sensation with high spatial-temporal resolution using large-aperture airborne ultrasound phased array, in World Haptics Conference (WHC) (Daejeon), 31–36.

[B117] Haynes A. Simons M. F. Helps T. Nakamura Y. Rossiter J. (2019). A wearable skin-stretching tactile interface for Human–Robot and Human–Human communication. IEEE Robot. Autom. Lett. 4, 1641–1646. 10.1109/LRA.2019.2896933

[B118] High C. M. McHugh H. F. Mills S. C. Amano S. Freund J. E. Vallabhajosula S. (2018). Vibrotactile feedback alters dynamics of static postural control in persons with parkinson's disease but not older adults at high fall risk. Gait Posture 63, 202–207. 10.1016/j.gaitpost.2018.05.010 29772496

[B119] Hill V. A. Fisher T. Schmid A. A. Crabtree J. Page S. J. (2014). Relationship between touch sensation of the affected hand and performance of valued activities in individuals with chronic stroke. Top. Stroke Rehabil. 21, 339–346. 10.1310/tsr2104-339 25150666

[B120] Holden J. K. Nguyen R. H. Francisco E. M. Zhang Z. Dennis R. G. Tommerdahl M. (2012). A novel device for the study of somatosensory information processing. J. Neurosci. Methods 204, 215–220. 10.1016/j.jneumeth.2011.11.007 22155443 PMC3413449

[B121] Horak F. B. (2006). Postural orientation and equilibrium: what do we need to know about neural control of balance to prevent falls? Age Ageing 35(Suppl. 2), ii7–ii11. 10.1093/ageing/afl077 16926210

[B122] Howard T. Marchal M. Lécuyer A. Pacchierotti C. (2020). PUMAH: pan-tilt ultrasound mid-air haptics for larger interaction workspace in virtual reality. IEEE Trans. Haptics 13, 38–44. 10.1109/TOH.2019.2963028 31902770

[B123] Huisman G. Bruijnes M. Kolkmeier J. Jung M. Frederiks A. D. Rybarczyk Y. (2013). Touching virtual agents: embodiment and mind, in International Summer Workshop on Multimodal Interfaces (Lisbon), 114–138.

[B124] Huisman G. Frederiks A. D. van Erp J. B. Heylen D. K. (2016). Simulating affective touch: Using a vibrotactile array to generate pleasant stroking sensations, in International Conference on Human Haptic Sensing and Touch Enabled Computer Applications (London), 240–250.

[B125] Hur P. Pan Y. DeBuys C. (2019). Free energy principle in human postural control system: Skin stretch feedback reduces the entropy. Sci. Rep. 9:16870. 10.1038/s41598-019-53028-1 31727928 PMC6856340

[B126] Israr A. Abnousi F. (2018). Towards pleasant touch: vibrotactile grids for social touch interactions, in Extended Abstracts of the 2018 CHI Conference on Human Factors in Computing Systems (Montréal, QC), 1–6.

[B127] Jacobs R. Brånemark R. Olmarker K. Rydevik B. Steenberghe D. van Brånemark, P. (2000). Evaluation of the psychophysical detection threshold level for vibrotactile and pressure stimulation of prosthetic limbs using bone anchorage or soft tissue support. Prosthet. Orthot. Int. 24, 133–142. 10.1080/03093640008726536 11061200

[B128] Jacobson G. P. Newman C. W. (1990). The development of the dizziness handicap inventory. Arch. Otolaryngol. Head Neck Surg. 116, 424–427. 2317323 10.1001/archotol.1990.01870040046011

[B129] Jaffe D. L. Brown D. A. Pierson-Carey C. D. Buckley E. L. Lew H. L. (2004). Stepping over obstacles to improve walking in individuals with poststroke hemiplegia. J. Rehabil. Res. Dev. 41, 283–292. 10.1682/jrrd.2004.03.0283 15543446

[B130] Jansen Y. Karrer T. Borchers J. (2010). MudPad: localized tactile feedback on touch surfaces, in Adjunct Proceedings of the 23nd Annual ACM Symposium on User Interface Software and Technology, 385–386.

[B131] Janssen L. J. F. Verhoeff L. L. Horlings C. G. C. Allum J. H. J. (2009). Directional effects of biofeedback on trunk sway during gait tasks in healthy young subjects. Gait Posture 29, 575–581. 10.1016/j.gaitpost.2008.12.009 19157877

[B132] Jiang L. Cutkosky M. R. Ruutiainen J. Raisamo R. (2009). Using haptic feedback to improve grasp force control in multiple sclerosis patients. IEEE Trans. Robot. 25, 593–601. 10.1109/TRO.2009.2019789

[B133] Johansson R. S. Flanagan J. R. (2009). Coding and use of tactile signals from the fingertips in object manipulation tasks. Nat. Rev. Neurosci. 10, 345–359. 10.1038/nrn2621 19352402

[B134] Johnson K. O. Yoshioka T. Vega-Bermudez F. (2000). Tactile functions of mechanoreceptive afferents innervating the hand. J. Clin. Neurophysiol. 17, 539–558. 10.1097/00004691-200011000-00002 11151974

[B135] Jones L. A. Sarter N. B. (2008). Tactile displays: guidance for their design and application. Hum. Factors 50, 90–111. 10.1518/001872008X250638 18354974

[B136] Jungmann M. Schlaak H. F. (2002). Miniaturised electrostatic tactile display with high structural compliance, in Proceedings of Eurohaptics (Edingburgh), 12–17.

[B137] Kajimoto H. Kawakami N. Maeda T. Tachi S. (2001). Electro-tactile display with force feedback. In Proc. World Multiconference on Systemics, Cybernetics and Informatics (SCI2001), 11, 95-99.

[B138] Kanjanapas S. Nunez C. M. Williams S. R. Okamura A. M. Luo M. (2019). Design and analysis of pneumatic 2-DoF soft haptic devices for shear display. IEEE Robot. Autom. Lett. 4, 1365–1371. 10.1109/LRA.2019.2895890

[B139] Kato G. Kuroda Y. Nisky I. Kiyokawa K. Takemura H. (2016). Design and Psychophysical Evaluation of the HapSticks: a novel non-grounded mechanism for presenting tool-mediated vertical forces. IEEE Trans. Haptics 10, 338–349. 10.1109/TOH.2016.2636824 27992349

[B140] Kaul O. B. Rohs M. (2017). HapticHead: a spherical vibrotactile grid around the head for 3D guidance in virtual and augmented reality, in Proceedings of the CHI Conference on Human Factors in Computing Systems (Denver, CO), 3729–3740.

[B141] Kern T. A. (2009). Engineering Haptic Devices: A Beginner's Guide for Engineers. New York, NY: Springer-Verlag. 10.1007/978-3-540-88248-0

[B142] Khaodhiar L. Niemi J. B. Earnest R. Lima C. Harry J. D. Veves A. (2003). Enhancing sensation in diabetic neuropathic foot with mechanical noise. Diabetes Care 26, 3280–3283. 10.2337/diacare.26.12.3280 14633814

[B143] Kim K. Colgate J. E. (2012). Haptic feedback enhances grip force control of sEMG-controlled prosthetic hands in targeted reinnervation amputees. IEEE Trans. Neural Syst. Rehabil. Eng. 20, 798–805. 10.1109/TNSRE.2012.2206080 22855230

[B144] Kim S. Kim C. Yang G. Yang T. Han B. Kang S.. (2009). Small and lightweight tactile display(SaLT) and its application, in World Haptics -Third Joint EuroHaptics conference and Symposium on Haptic Interfaces for Virtual Environment and Teleoperator Systems (Salt Lake City, UT), 69–74.

[B145] Kim S. Kim P. Park C. Choi S. (2016). A new tactile device using magneto-rheological sponge cells for medical applications: experimental investigation. Sensors Actuat. A Phys. 239, 61–69. 10.1016/j.sna.2016.01.016

[B146] King D. A. Hume P. Tommerdahl M. (2018). Use of the Brain-Gauge somatosensory assessment for monitoring recovery from concussion: a case study. J. Physiother. Res. 2:13.

[B147] Koehler M. Usevitch N. S. Okamura A. M. (2020). Model-based design of a soft 3-D haptic shape display. IEEE Trans. Robot. 36, 613–628. 10.1109/TRO.2020.2980114

[B148] Koh G. C. Yen S. C. Tay A. Cheong A. Ng Y. S. De Silva D. A.. (2015). Singapore tele-technology aided rehabilitation in stroke (STARS) trial: protocol of a randomized clinical trial on tele-rehabilitation for stroke patients. BMC Neurol. 15:161. 10.1186/s12883-015-0420-3 26341358 PMC4560876

[B149] Krueger A. R. Giannoni P. Shah V. Casadio M. Scheidt R. A. (2017). Supplemental vibrotactile feedback control of stabilization and reaching actions of the arm using limb state and position error encodings. J. Neuroeng. Rehabil. 14:69. 10.1186/s12984-017-0281-7 28693522 PMC5504615

[B150] Kumar N. A. Yoon H. U. Hur P. (2017). A user-centric feedback device for powered wheelchairs comprising a wearable skin stretch device and a haptic joystick, in IEEE Workshop on Advanced Robotics and its Social Impacts (ARSO) (Austin, TX), 1–2.

[B151] Kuniyasu Y. Sato M. Fukushima S. Kajimoto H. (2012). Transmission of forearm motion by tangential deformation of the skin, in Proceedings of the 3rd Augmented Human International Conference (Megève), 1–4.

[B152] Kyung K. U. Park J. S. (2007). Ubi-Pen: Development of a compact tactile display module and its application to a haptic stylus, in Second Joint EuroHaptics Conference and Symposium on Haptic Interfaces for Virtual Environment and Teleoperator Systems (WHC'07) (Tsukaba), 109–114.

[B153] Lai S. Ahmed U. Bollineni A. Lewis R. Ramchandren S. (2014). Diagnostic accuracy of qualitative vs. quantitative tuning forks: outcome measure for neuropathy. J. Clin. Neuromuscul. Dis. 15:96. 10.1097/CND.0000000000000019 24534830 PMC4957578

[B154] Lanting S. M. Spink M. J. Tehan P. E. Vickers S. Casey S. L. Chuter V. H. (2020). Non-invasive assessment of vibration perception and protective sensation in people with diabetes mellitus: inter-and intra-rater reliability. J. Foot Ankle Res. 13, 1–7. 10.1186/s13047-020-0371-9 31988664 PMC6966840

[B155] Laufer Y. Elboim-Gabyzon M. (2011). Does sensory transcutaneous electrical stimulation enhance motor recovery following a stroke? A systematic review. Neurorehabil. Neural Repair 25, 799–809. 10.1177/1545968310397205 21746874

[B156] Lederman S. J. Klatzky R. L. (2009). Haptic perception: a tutorial. Attent. Percept. Psychophys. 71, 1439–1459. 10.3758/APP 19801605

[B157] Lee B. Fung A. Thrasher T. A. (2018). The effects of coding schemes on vibrotactile biofeedback for dynamic balance training in parkinson's disease and healthy elderly individuals. IEEE Trans. Neural Syst. Rehabil. Eng. 26, 153–160. 10.1109/TNSRE.2017.2762239 29053448

[B158] Lee B. C. Martin B. J. Sienko K. H. (2012). Directional postural responses induced by vibrotactile stimulations applied to the torso. Exp. Brain Res. 222, 471–482. 10.1007/s00221-012-3233-2 22968737

[B159] Leo F. Cocchi E. Brayda L. (2016). The effect of programmable tactile displays on spatial learning skills in children and adolescents of different visual disability. IEEE Trans. Neural Syst. Rehabil. Eng. 25, 861–872. 10.1109/TNSRE.2016.2619742 27775905

[B160] Leonardis D. Solazzi M. Bortone I. Frisoli A. (2017). A 3-RSR haptic wearable device for rendering fingertip contact forces. IEEE Trans. Haptics 10, 305–316. 10.1109/TOH.2016.2640291 28113306

[B161] Lieberman J. Breazeal C. (2007). Development of a wearable vibrotactile feedback suit for accelerated human motor learning, in Proceedings IEEE International Conference on Robotics and Automation (Roma), 4001–4006.

[B162] Liu W. Lipsitz L. A. Montero-Odasso M. Bean J. Kerrigan D. C. Collins J. J. (2002). Noise-enhanced vibrotactile sensitivity in older adults, patients with stroke, and patients with diabetic neuropathy. Arch. Phys. Med. Rehabil. 83, 171–176. 10.1053/apmr.2002.28025 11833019

[B163] Long B. Seah S. A. Carter T. Subramanian S. (2014). Rendering volumetric haptic shapes in mid-air using ultrasound. ACM Trans. Graph 33, 1–10. 10.1145/2661229.2661257

[B164] Ma C. Z. H. Lee W. C. C. (2017). A wearable vibrotactile biofeedback system improves balance control of healthy young adults following perturbations from quiet stance. Hum. Mov. Sci, 55, 54–60. 10.1016/j.humov.2017.07.006 28763702

[B165] Magalhães F. H. Kohn A. F. (2011). Vibratory noise to the fingertip enhances balance improvement associated with light touch. Exp. Brain Res. 209, 139–151. 10.1007/s00221-010-2529-3 21191573

[B166] Magill R. A. (2004). Motor Learning and Control: Concepts and Applications, 7th Edn. New York, NY: McGraw-Hill.

[B167] Maki B. E. McIlroy W. E. (1997). The role of limb movements in maintaining upright stance: the “change-in-support” strategy. Phys. Ther. 77, 488–507. 10.1093/ptj/77.5.488 9149760

[B168] Makino Y. Furuyama Y. Inoue S. Shinoda H. (2016). HaptoClone (Haptic-Optical Clone) for mutual tele-environment by real-time 3D image transfer with midair force feedback, in CHI (San Jose, CA), 1980–1990.

[B169] McKinney Z. Heberer K. Nowroozi B. N. Greenberg M. Fowler E. Grundfest W. (2014). Pilot evaluation of wearable tactile biofeedback system for gait rehabilitation in peripheral neuropathy, in 2014 IEEE Haptics Symposium (HAPTICS) (Houston, TX: IEEE), 135–140. 24732521

[B170] Meli L. Hussain I. Aurilio M. Malvezzi M. O'Malley M. K. Prattichizzo D. (2018). The hBracelet: a wearable haptic device for the distributed mechanotactile stimulation of the upper limb. IEEE Robot. Autom. Lett. 3, 2198–2205. 10.1109/LRA.2018.2810958

[B171] Merrett G. V. Metcalf C. D. Zheng D. Cunningham S. Barrow S. Demain S. H. (2011). Design and qualitative evaluation of tactile devices for stroke rehabilitation, in IET Seminar on Assisted Living (London), 1–6. 10.1049/ic.2011.0025

[B172] Mikkelsen M. He J. Tommerdahl M. Edden R. A. Mostofsky S. H. Puts N. A. (2020). Reproducibility of flutter-range vibrotactile detection and discrimination thresholds. Sci. Rep. 10, 1–14. 10.1038/s41598-020-63208-z 32300187 PMC7162987

[B173] Minamizawa K. Fukamachi S. Kajimoto H. Kawakami N. Tachi S. (2007). Gravity grabber: wearable haptic display to present virtual mass sensation, in ACM SIGGRAPH 2007 Emerging Technologies (San Diego, CA), 8-es.

[B174] Minamizawa K. Kamuro S. Fukamachi S. Kawakami N. Tachi S. (2008). GhostGlove: haptic existence of the virtual world, in ACM SIGGRAPH 2008 New Tech Demos (Los Angeles, CA), 18.

[B175] Monnai Y. Hasegawa K. Fujiwara M. Yoshino K. Inoue S. Shinoda H. (2014). HaptoMime: mid-air haptic interaction with a floating virtual screen, in User Interface Software and Technology Symposium (UIST) (Honolulu, HA), 663–667. 10.1145/2642918.2647407

[B176] Monnai Y. Hasegawa K. Fujiwara M. Yoshino K. Inoue S. Shinoda H. (2015). Adding texture to aerial images using ultrasounds, in Haptic Interaction (Tokyo: Springer), 59–61.

[B177] Moseley G. L. Zalucki N. M. Wiech K. (2008). Tactile discrimination, but not tactile stimulation alone, reduces chronic limb pain. Pain 137, 600–608. 10.1016/j.pain.2007.10.021 18054437

[B178] Moss F. Ward L. M. Sannita W. G. (2004). Stochastic resonance and sensory information processing: a tutorial and review of application. Clin. Neurophysiol. 115, 267–281. 10.1016/j.clinph.2003.09.014 14744566

[B179] Muijzer-Witteveen H. J. B. Nataletti S. Agnello M. Casadio M. Van Asseldonk E. H. F. (2017). Vibrotactile feedback to control the amount of weight shift during walking - a first step towards better control of an exoskeleton for spinal cord Injury subjects, in IEEE International Conference on Rehabilitation Robotics (London: IEEE), 1482–1487. 10.1109/ICORR.2017.8009457 28814029

[B180] Mullenbach J. Shultz C. Colgate J. E. Piper A. M. (2014). Exploring affective communication through variable-friction surface haptics, in Proceedings of the SIGCHI Conference on Human Factors in Computing Systems (Toronto, ON), 3963–3972.

[B181] Muramatsu Y. Niitsuma M. Thomessen T. (2012). Perception of tactile sensation using vibrotactile glove interface, in 2012 IEEE 3rd International Conference on Cognitive Infocommunications (CogInfoCom) (Kosice: IEEE), 621–626.

[B182] Nakamura M. Jones L. (2003). An actuator for the tactile vest - a torso-based haptic device, in 11th Symposium on Haptic Interfaces for Virtual Environment and Teleoperator Systems. HAPTICS. Proceedings (Los Angeles, CA), 333–339.

[B183] Nanhoe-Mahabier W. Allum J. H. Pasman E. P. Overeem S. Bloem B. R. (2012). The effects of vibrotactile biofeedback training on trunk sway in Parkinson's disease patients. Parkinson. Relat. Disord. 18, 1017–1021. 10.1016/j.parkreldis.2012.05.018 22721975

[B184] Navarro E. González P. López-Jaquero V. Montero F. Molina J. P. Romero-Ayuso D. (2018). Adaptive, multisensorial, physiological and social: the next generation of telerehabilitation systems. Front. Neuroinform. 12:43. 10.3389/fninf.2018.00043 30042671 PMC6049338

[B185] Nitsch V. Färber B. (2012). A meta-analysis of the effects of haptic interfaces on task performance with teleoperation systems. IEEE Trans. Haptics 6, 387–398. 10.1109/TOH.2012.62 24808391

[B186] Nobusako S. Osumi M. Matsuo A. Furukawa E. Maeda T. Shimada S.. (2019). Subthreshold vibrotactile noise stimulation immediately improves manual dexterity in a child with developmental coordination disorder: a single-case study. Front. Neurol. 10:717. 10.3389/fneur.2019.00717 31312179 PMC6614204

[B187] Norman S. L. Doxon A. J. Gleeson B. T. Provancher W. R. (2014). Planar hand motion guidance using fingertip skin-stretch feedback. IEEE Trans. Haptics 7, 121–130. 10.1109/TOH.2013.2296306 24968376

[B188] Norrsell U. Olausson H. (1992). Human, tactile, directional sensibility and its peripheral origins. Acta Physiol. Scand. 144, 155–161. 1575049 10.1111/j.1748-1716.1992.tb09280.x

[B189] Nunez C. M. Huerta B. N. Okamura A. M. Culbertson H. (2020). Investigating social haptic illusions for tactile stroking (SHIFTS), in IEEE Haptics Symposium (HAPTICS), 629–636.

[B190] Nunez C. M. Williams S. R. Okamura A. M. Culbertson H. (2019). Understanding continuous and pleasant linear sensations on the forearm from a sequential discrete lateral skin-slip haptic device. IEEE Trans. Haptics 12, 414–427. 10.1109/TOH.2019.2941190 31536015

[B191] Obrist M. Subramanian S. Gatti E. Long B. Carter T. (2015). Emotions mediated through mid-air haptics, in Proceedings of the 33rd Annual ACM Conference on Human Factors in Computing Systems (Seoul), 2053–2062.

[B192] Omori R. Kuroda Y. Yoshimoto S. Oshiro O. (2019). A wearable skin stretch device for lower limbs: investigation of curvature effect on slip, in IEEE World Haptics Conference (WHC) (Tokyo), 37–42. 10.1109/WHC.2019.8816129

[B193] O'Neill J. McCann S. M. Lagan K. M. (2006). Tuning fork (128 Hz) versus neurothesiometer: a comparison of methods of assessing vibration sensation in patients with diabetes mellitus. Int. J. Clin. Pract. 60, 174–178. 10.1111/j.1742-1241.2005.00650.x 16451290

[B194] Pacchierotti C. Salvietti G. Hussain I. Meli L. Prattichizzo D. (2016). The hRing: a wearable haptic device to avoid occlusions in hand tracking, in Presented at the IEEE Haptics Symposium (HAPTICS) (Philadelphia, PA), 134–139. 10.1109/HAPTICS.2016.7463167

[B195] Pacchierotti C. Sinclair S. Solazzi M. Frisoli A. Hayward V. Prattichizzo D. (2017). Wearable haptic systems for the fingertip and the hand: taxonomy, review, and perspectives. IEEE Trans. Haptics 10, 580–600. 10.1109/TOH.2017.2689006 28500008

[B196] Pan Y. T. Yoon H. U. Hur P. (2017). A portable sensory augmentation device for balance rehabilitation using fingertip skin stretch feedback. IEEE Trans. Neural Syst. Rehabil. Eng. 25, 31–39. 10.1109/TNSRE.2016.2542064 26992163

[B197] Paré M. Carnahan H. Smith A. (2002). Magnitude estimation of tangential force applied to the fingerpad. Exp. Brain Res. 142, 342–348. 10.1007/s00221-001-0939-y 11819042

[B198] Pasquero J. Hayward V. (2003). STReSS: a practical tactile display system with one millimeter spatial resolution and 700 Hz refresh rate, in Proc. Eurohaptics (Dublin), 94–110.

[B199] Patel S. Park H. Bonato P. Chan L. Rodgers M. (2012). A review of wearable sensors and systems with application in rehabilitation. J. Neuroeng. Rehabil. 9:21. 10.1186/1743-0003-9-21 22520559 PMC3354997

[B200] Pearson K. G. (2000). Plasticity of neuronal networks in the spinal cord: modifications in response to altered sensory input. Prog. Brain Res. 128, 61–70. 10.1016/S0079-6123(00)28007-2 11105669

[B201] Perez M. A. Field-Fote E. C. Floeter M. K. (2003). Patterned sensory stimulation induces plasticity in reciprocal ia inhibition in humans. J. Neurosci. 23, 2014–2018. 10.1523/JNEUROSCI.23-06-02014.2003 12657659 PMC6742007

[B202] Perkins B. A. Olaleye D. Zinman B. Bril V. (2001). Simple screening tests for peripheral neuropathy in the diabetes clinic. Diabetes Care 24, 250–256. 10.2337/diacare.24.2.250 11213874

[B203] Pezent E. Fani S. Clark J. Bianchi M. O'Malley M. K. (2019). Spatially separating haptic guidance from task dynamics through wearable devices. IEEE Trans. Haptics 12, 581–593. 10.1109/TOH.2019.2919281 31144646

[B204] Prattichizzo D. Chinello F. Pacchierotti C. Malvezzi M. (2013). Towards wearability in fingertip haptics: a 3-DoF Wearable Device for Cutaneous Force Feedback. IEEE Trans. Haptics 6, 506–516. 10.1109/TOH.2013.53 24808402

[B205] Prattichizzo D. Chinello F. Pacchierotti C. Minamizawa K. (2010). RemoTouch: a system for remote touch experience, in Presented at the 19th International Symposium in Robot and Human Interactive Communication (Viareggio), 676–679. 10.1109/ROMAN.2010.5598606

[B206] Provancher W. R. Cutkosky M. R. Kuchenbecker K. J. Niemeyer G. (2005). Contact location display for haptic perception of curvature and object motion. Int. J. Robot. Res., 24, 691–702. 10.1177/0278364905057121

[B207] Provancher W. R. Sylvester N. D. (2009). Fingerpad skin stretch increases the perception of virtual friction. IEEE Trans. Haptics 2, 212–223. 10.1109/TOH.2009.34 27788106

[B208] Puts N. A. J. Edden R. A. E. Wodka E. L. Mostofsky S. H. Tommerdahl M. (2013). A vibrotactile behavioral battery for investigating somatosensory processing in children and adults. J. Neurosci. Methods 218, 39–47. 10.1016/j.jneumeth.2013.04.012 23660524 PMC4106128

[B209] Quek Z. F. Schorr S. B. Nisky I. Okamura A. M. Provancher W. R. (2013). Sensory augmentation of stiffness using fingerpad skin stretch, in Presented at the World Haptics Conference (WHC) (Daejeon), 467–472. 10.1109/WHC.2013.6548453

[B210] Quek Z. F. Schorr S. B. Nisky I. Okamura A. M. Provancher W. R. (2014a). Augmentation of stiffness perception with a 1-degree-of-freedom skin stretch device. IEEE Transactions on Human-Machine Systems 44, 731–742. 10.1109/THMS.2014.2348865

[B211] Quek Z. F. Schorr S. B. Nisky I. Provancher W. R. Okamura A. M. (2014b). Sensory substitution using 3-degree-of-freedom tangential and normal skin deformation feedback, in Pesented at the IEEE Haptics Symposium (HAPTICS) (Houston, TX), 27–33. 10.1109/HAPTICS.2014.6775429 25647582

[B212] Quek Z. F. Schorr S. B. Nisky I. Provancher W. R. Okamura A. M. (2015a). Sensory substitution and augmentation using 3-degree-of-freedom skin deformation feedback. IEEE Trans. Haptics 8, 209–221. 10.1109/TOH.2015.2398448 25647582

[B213] Quek Z. F. Schorr S. B. Nisky I. Provancher W. R. Okamura A. M. (2015b). Sensory substitution of force and torque using 6-DoF tangential and normal skin deformation feedback, Presented at the IEEE International Conference on Robotics and Automation (ICRA) (Seattle, WA), 264–271. 10.1109/ICRA.2015.7139010

[B214] Raitor M. Walker J. M. Okamura A. M. Culbertson H. (2017). WRAP: Wearable, restricted-aperture pneumatics for haptic guidance, in 2017 IEEE International Conference on Robotics and Automation (ICRA) (Singapore), 427–432.

[B215] Rakkolainen I. Sand A. Raisamo R. (2019). A survey of mid-air ultrasonic tactile feedback, in IEEE International Symposium on Multimedia (ISM) (San Diego, CA), 10.1109/ISM46123.2019.00022

[B216] Rinderknecht M. D. Dueñas J. A. Held J. P. Lambercy O. Conti F. M. Zizlsperger L.. (2019). Automated and quantitative assessment of tactile mislocalization after stroke. Front. Neurol. 10:593. 10.3389/fneur.2019.00593 31244757 PMC6581709

[B217] Rinderknecht M. D. Gross R. Leuenberger K. Lambercy O. Gassert R. (2015). Objective assessment of vibrotactile mislocalization using a haptic glove, in IEEE International Conference on Rehabilitation Robotics (ICORR) (Singapore), 145–150.

[B218] Risi N. Shah V. Mrotek L. A. Casadio M. Scheidt R. A. (2019). Supplemental vibrotactile feedback of real-time limb position enhances precision of goal-directed reaching. J. Neurophysiol. 122, 22–38. 10.1152/jn.00337.2018 30995149 PMC6689770

[B219] Roger J. Darfour D. Dha A. Hickman O. Shaubach L. Shepard K. (2002). Physiotherapists' use of touch in inpatient settings. Physiother. Res. Int. 7, 170–186. 10.1002/pri.253 12426914

[B220] Romanus T. Frish S. Maksymenko M. Frier W. Corenthy L. Georgiou O. (2019). Mid-air haptic bio-holograms in mixed reality, in Adjunct Proceedings of the 2019 IEEE International Symposium on Mixed and Augmented Reality, ISMAR-Adjunct 2019 (Beijing). 10.1109/ISMAR-Adjunct.2019.00-14

[B221] Rose T. Nam C. S. Chen K. B. (2018). Immersion of virtual reality for rehabilitation-Review. Appl. Ergon. 69, 153–161. 10.1016/j.apergo.2018.01.009 29477323

[B222] Rossi-Izquierdo M. Ernst A. Soto-Varela A. Santos-Pérez S. Faraldo-García A. Sesar-Ignacio A.. (2013). Vibrotactile neurofeedback balance training in patients with parkinson's disease: reducing the number of falls. Gait Posture 37, 195–200. 10.1016/j.gaitpost.2012.07.002 22841586

[B223] Rotella M. F. Guerin K. He X. Okamura A. M. (2012). Hapi bands: a haptic augmented posture interface, in IEEE Haptics Symposium (HAPTICS) (Vancouver, BC), 163–170.

[B224] Rutten E. Van Den Bogaert L. Geerts D. (2020). From initial encounter with mid-air haptic feedback to repeated use: the role of the novelty effect in user experience. IEEE Trans. Haptics. 10.1109/TOH.2020.3043658. [Epub ahead of print]. 33296309

[B225] Sarakoglou I. Tsagarakis N. Caldwell D. G. (2005). A portable fingertip tactile feedback array - transmission system reliability and modelling, in First Joint Eurohaptics Conference and Symposium on Haptic Interfaces for Virtual Environment and Teleoperator Systems. World Haptics Conference (Pisa), 547–548.

[B226] Schorr S. B. Okamura A. (2017a). Three-dimensional skin deformation as force substitution: Wearable device design and performance during haptic exploration of virtual environments. IEEE Trans. Haptics. 10, 418–430. 10.1109/TOH.2017.2672969 28237933

[B227] Schorr S. B. Okamura A. M. (2017b). Fingertip tactile devices for virtual object manipulation and exploration, in Proceedings of the 2017 CHI Conference on Human Factors in Computing Systems (Denver), 3115–3119.

[B228] Schorr S. B. Quek Z. F. Nisky I. Provancher W. R. Okamura A. M. (2015). Tactor-induced skin stretch as a sensory substitution method in teleoperated palpation. IEEE Trans. Hum. Mach. Syst. 45, 714–726. 10.1109/THMS.2015.2463090

[B229] Schorr S. B. Quek Z. F. Romano R. Y. Nisky I. Provancher W. R. Okamura A. M. (2013). Sensory substitution via cutaneous skin stretch feedback, Presented at the 2013 IEEE International Conference on Robotics and Automation (ICRA) (Karlsruhe), 2341–2346.

[B230] Schweisfurth M. A. Markovic M. Dosen S. Teich F. Graimann B. Farina D. (2016). Electrotactile EMG feedback improves the control of prosthesis grasping force. J. Neural Eng. 13:056010. 10.1088/1741-2560/13/5/056010 27547992

[B231] Seim C. E. Ritter B. Starner T. E. Flavin K. Lansberg M. G. Okamura A. M. (2020a). Perspectives on the design and performance of upper-limb wearable stimulation devices for stroke survivors with hemiplegia and spasticity. bioRxiv [Preprint]. 10.1101/2020.08.20.260000

[B232] Seim C. E. Wolf S. L. Starner T. E. (2020b). Wearable vibrotactile stimulation for upper extremity rehabilitation in chronic stroke: clinical feasibility trial using the VTS Glove. arXiv preprint arXiv:2007.09262. 33485371 10.1186/s12984-021-00813-7 PMC7824932

[B233] Semmes J. Weinstein S. Ghent L. Teuber H. L. (1960). Somatosensory Changes After Penetrating Brain Wounds in Man. Commonwealth Fund. Cambridge, MA: Harvard University Press.

[B234] Seo N. J. Enders L. R. Fortune A. Cain S. Vatinno A. A. Schuster E.. (2020). Phase I safety trial: extended daily peripheral sensory stimulation using a wrist-worn vibrator in stroke survivors. Transl. Stroke Res. 11, 204–213. 10.1007/s12975-019-00724-9 31444692 PMC7035973

[B235] Seo N. J. Kosmopoulos M. L. Enders L. R. Hur P. (2014). Effect of remote sensory noise on hand function post stroke. Front. Hum. Neurosci. 8:934. 10.3389/fnhum.2014.00934 25477806 PMC4235074

[B236] Seo N. J. Woodbury M. L. Bonilha L. Ramakrishnan V. Kautz S. A. Downey R. J.. (2019). TheraBracelet stimulation during task-practice therapy to improve upper extremity function after stroke: a pilot randomized controlled study. Phys. Ther. 99, 319–328. 10.1093/ptj/pzy143 30690609 PMC6383710

[B237] Serrada I. Hordacre B. Hillier S. L. (2019). Does sensory retraining improve sensation and sensorimotor function following stroke: a systematic review and meta-analysis. Front. Neurosci. 13, 402. 10.3389/fnins.2019.00402 31114472 PMC6503047

[B238] Shah V. A. Casadio M. Scheidt R. A. Mrotek L. A. (2019). Vibration propagation on the skin of the arm. Appl. Sci. 9:4329. 10.3390/app9204329 PMC8493869 34621542

[B239] Shah V. A. Risi N. Ballardini G. Mrotek L. A. Casadio M. Scheidt R. A. (2018). Effect of dual tasking on vibrotactile feedback guided reaching – a pilot study, in Haptics: Science, Technology, and Applications: 11th International Conference, EuroHaptics (Pisa), 10893, 3–14. 10.1007/978-3-319-93445-7_1 31179445 PMC6555617

[B240] Shakeri G. Williamson J. H. Brewster S. (2017). Novel multimodal feedback techniques for in-car mid-air gesture interaction, in AutomotiveUI 2017 - 9th International ACM Conference on Automotive User Interfaces and Interactive Vehicular Applications, Proceedings (Oldenburg). 10.1145/3122986.3123011

[B241] Shakeri G. Williamson J. H. Brewster S. (2018). May the force be with you: ultrasound haptic feedback for mid-air gesture interaction in cars, in Proceedings - 10th International ACM Conference on Automotive User Interfaces and Interactive Vehicular Applications (Toronto, ON: AutomotiveUI). 10.1145/3239060.3239081

[B242] Sherrick C. E. Rogers R. (1966). Apparent haptic movement. Percept. Psychophys. 1, 175–180. 10.3758/BF03215780

[B243] Shi S. Leineweber M. J. Andrysek J. (2019). Exploring the tactor configurations of vibrotactile feedback systems for use in lower-limb prostheses. J. Vibr. Acoust. 141:051009. 10.1115/1.4043610

[B244] Shimizu Y. Saida S. Shimura H. (1993). Tactile pattern recognition by graphic display: importance of 3-D information for haptic perception of familiar objects. Percept. Psychophys. 53, 43–48. 10.3758/BF03211714 8433905

[B245] Shull P. B. Damian D. D. (2015). Haptic wearables as sensory replacement, sensory augmentation and trainer - a review. J. Neuroeng. Rehabil. 12:59. 10.1186/s12984-015-0055-z 26188929 PMC4506766

[B246] Sienko K. H. Balkwill M. D. Oddsson L. I. E. Wall C. (2013). The effect of vibrotactile feedback on postural sway during locomotor activities. J. Neuroeng. Rehabil. 10:93. 10.1186/1743-0003-10-93 23938136 PMC3751349

[B247] Sienko K. H. Balkwill M. D. Wall C. (2012). Biofeedback improves postural control recovery from multi-axis discrete perturbations. J. Neuroeng. Rehabil. 9:53. 10.1186/1743-0003-9-53 22863399 PMC3477042

[B248] Simpson R. Robinson L. (2020). Rehabilitation after critical illness in people with COVID-19 infection. Am. J. Phys. Med. Rehabil. 99, 470–474. 10.1097/PHM.0000000000001443 32282359 PMC7253039

[B249] Smith C. Pezent E. O'Malley M. K. (2020). Spatially separated cutaneous haptic guidance for training of a virtual sensorimotor task, in Presented at the IEEE Haptics Symposium (HAPTICS) (Crystal City, VA), 974–979. 10.1109/HAPTICS45997.2020.ras.HAP20.11.2032900c

[B250] Sofia K. O. Jones L. (2013). Mechanical and psychophysical studies of surface wave propagation during vibrotactile stimulation. IEEE Trans. Haptics 6, 320–329. 10.1109/TOH.2013.1 24808328

[B251] Solazzi M. Provancher W. R. Frisoli A. Bergamasco M. (2011). Design of a SMA actuated 2-DoF tactile device for displaying tangential skin displacement, Presented at the IEEE World Haptics Conference (Istanbul), 31–36. 10.1109/WHC.2011.5945457

[B252] Standen P. J. Threapleton K. Connell L. Richardson A. Brown D. J. Battersby S.. (2015). Patients' use of a home-based virtual reality system to provide rehabilitation of the upper limb following stroke. Phys. Ther. 95, 350–359. 10.2522/ptj.20130564 25212521

[B253] Stanley A. A. Kuchenbecker K. J. (2012). Evaluation of tactile feedback methods for wrist rotation guidance. IEEE Trans. Haptics 5, 240–251. 10.1109/TOH.2012.33 26964110

[B254] Steihaug S. Lippestad J. Werner A. (2016). Between ideals and reality in home-based rehabilitation. Scand. J. Prim. Health Care 34, 46–54. 10.3109/02813432.2015.1132888 26828898 PMC4911023

[B255] Stephens-Fripp B. Mutlu R. Alici G. (2018). Applying mechanical pressure and skin stretch simultaneously for sensory feedback in prosthetic hands, in 7th IEEE International Conference on Biomedical Robotics and Biomechatronics (Biorob) (Enschede), 230–235.

[B256] Stevens J. C. (1982). Temperature can sharpen tactile acuity. Percept. Psychophys. 31, 577–580. 10.3758/BF03204192 7122194

[B257] Strong R. Gaver B. (1996). Feather, scent and shaker: supporting simple intimacy. Proc. CSCW 96, 29–30.

[B258] Sullivan J. E. Hedman L. D. (2008). Sensory dysfunction following stroke: incidence, significance, examination, and intervention. Top. Stroke Rehabil. 15, 200–217. 10.1310/tsr1503-200 18647725

[B259] Sullivan J. L. Dunkelberger N. Bradley J. Young J. Israr A. Lau F.. (2019). Multi-sensory stimuli improve distinguishability of cutaneous haptic cues. IEEE Trans. Haptics? 13, 286–297. 10.1109/TOH.2019.2922901 31217130

[B260] Suzuki S. Takahashi R. Nakajima M. Hasegawa K. Makino Y. Shinoda H. (2018). Midair haptic display to human upper body, in Proc. Soc. Instrument and Control Engineers Japan (SICE'18) (Nara), 848–853.

[B261] Svensson P. Wijk U. Björkman A. Antfolk C. (2017). A review of invasive and non-invasive sensory feedback in upper limb prostheses. Expert Rev. Med. Dev. 14, 439–447. 10.1080/17434440.2017.1332989 28532184

[B262] Sylvester N. D. Provancher W. R. (2007). Effects of longitudinal skin stretch on the perception of friction, in Second Joint EuroHaptics Conference and Symposium on Haptic Interfaces for Virtual Environment and Teleoperator Systems (WHC'07) (Tsukaba), 373–378.

[B263] Takeuchi T. Futatsuka M. Imanishi H. Yamada S. (1986). Pathological changes observed in the finger biopsy of patients with vibration-induced white finger. Scand. J. Work Environ. Health 12, 280–283. 10.5271/sjweh.2140 3775312

[B264] Talhan A. Jeon S. (2018). Pneumatic actuation in haptic-enabled medical simulators: a review. IEEE Access 6, 3184–3200. 10.1109/ACCESS.2017.2787601

[B265] Taylor M. W. Taylor J. L. Seizova-Cajic T. (2017). Muscle vibration-induced illusions: review of contributing factors, taxonomy of illusions and user's guide. Multisens. Res. 30, 25–63. 10.1163/22134808-00002544

[B266] Taylor P. M. Hosseini-Sianaki A. Varley C. J. Pollet D. M. (1997). Advances in an Electrorheological Fluid Based Tactile Array. IEE Colloquium on Developments in Tactile Display. London: IET.

[B267] Tenforde A. S. Borgstrom H. Polich G. Steere H. Davis I. S. Cotton K.. (2020). Outpatient physical, occupational, and speech therapy synchronous telemedicine: a survey study of patient satisfaction with virtual visits during the COVID-19 pandemic. Am. J. Phys. Med. Rehabil. 99, 977–981. 10.1097/PHM.0000000000001571 32804713 PMC7526401

[B268] Thomas P. Baldwin C. Bissett B. Boden I. Gosselink R. Granger C. L.. (2020). Physiotherapy management for COVID-19 in the acute hospital setting: clinical practice recommendations. J. Physiother. 66, 73–82. 10.1016/j.jphys.2020.03.011 32312646 PMC7165238

[B269] Tommerdahl M. Lensch R. Francisco E. Holden J. Favorov O. (2019). The brain gauge: a novel tool for assessing brain health. J. Sci. Med. 1, 1–19. 10.37714/josam.v1i1.4

[B270] Tsalamlal M. Y. Ouarti N. Ammi M. (2013). Psychophysical study of air jet based tactile stimulation, in World Haptics Conference (WHC) (Daejeon), 639–644.

[B271] Tsetserukou D. Hosokawa S. Terashima K. (2014). LinkTouch: a wearable haptic device with five-bar linkage mechanism for presentation of two-DOF force feedback at the fingerpad, in IEEE Haptics Symposium (HAPTICS) (Houston, TX), 307–312.

[B272] Turville M. L. Cahill L. S. Matyas T. A. Blennerhassett J. M. Carey L. M. (2019). The effectiveness of somatosensory retraining for improving sensory function in the arm following stroke: a systematic review. Clin. Rehabil. 33, 834–846. 10.1177/0269215519829795 30798643

[B273] Van Breda E. Verwulgen S. Saeys W. Wuyts K. Peeters T. Truijen S. (2017). Vibrotactile feedback as a tool to improve motor learning and sports performance: a systematic review. BMJ Open Sport Exerc. Med. 3:e000216. 10.1136/bmjsem-2016-000216 28761708 PMC5530110

[B274] Van Erp Jan B. F. Van Veen Hendrik A. H. C. Jansen C. Dobbins T. (2005). Waypoint navigation with a vibrotactile waist belt. ACM Trans. Appl. Percept. 2, 106–117. 10.1145/1060581.1060585

[B275] Van Vliet P. M. Wulf G. (2006). Extrinsic feedback for motor learning after stroke: what is the evidence? Disabil. Rehabil. 28, 831–840. 10.1080/09638280500534937 16777770

[B276] Vo D. Brewster S. A. (2015). Touching the invisible: localizing ultrasonic haptic cues, in IEEE World Haptics Conference (WHC) (Evanston, IL), 368–373.

[B277] Wall C. Weinberg M. S. Schmidt P. B. Krebs D. E. (2001). Balance prosthesis based on micromechanical sensors using vibrotactile feedback of tilt. IEEE Trans. Biomed. Eng. 48, 1153–1161. 10.1109/10.951518 11585039

[B278] Wall P. D. Noordenbos W. (1977). Sensory functions which remain in man after complete transection of dorsal columns. Brain 100, 641–653. 564735 10.1093/brain/100.4.641

[B279] Wan A. H. Wong D. W. Ma C. Z. Zhang M. Lee W. C. (2016). Wearable vibrotactile biofeedback device allowing identification of different floor conditions for lower-limb amputees. Arch. Phys. Med. Rehabil. 97, 1210–1213. 10.1016/j.apmr.2015.12.016 26763948

[B280] Wang C. Huang D. Y. Hsu S. W. Lin C. L. Chiu Y. L. Hou C. E.. (2020). Gaiters: exploring skin stretch feedback on legs for enhancing virtual reality experiences, in Proceedings of the CHI Conference on Human Factors in Computing Systems (Honolulu, HI), 1–14.

[B281] Wang Q. Markopoulos P. Yu B. Chen W. Timmermans A. (2017). Interactive wearable systems for upper body rehabilitation: a systematic review. J. Neuroeng. Rehabil. 14, 1–21. 10.1186/s12984-017-0229-y 28284228 PMC5346195

[B282] Weisenberger J. M. Broadstone S. M. Saunders F. A. (1989). Evaluation of two multichannel tactile aids for the hearing impaired. J. Acoust. Soc. Am. 86, 1764–1775. 2808925 10.1121/1.398608

[B283] Westebring van der Putten E. P. van den Dobbelsteen J. J. Goossens R. H. M. Jakimowicz J. J. Dankelman J. (2010). The effect of augmented feedback on grasp force in laparoscopic grasp control. IEEE Trans. Haptics 3, 280–291. 10.1109/TOH.2010.23 27788113

[B284] Winstein C. J. Schmidt R. A. (1990). Reduced frequency of knowledge of results enhances motor skill learning. J. Exp. Psychol. Learn. Mem. Cogn. 16, 677–691. 10.1037/0278-7393.16.4.677

[B285] Wu S. W. Fan R. E. Wottawa C. R. Fowler E. G. Bisley J. W. Grundfest W. S.. (2010). Torso-based tactile feedback system for patients with balance disorders, in 2010 IEEE Haptics Symposium (Waltham, MA), 359–362.

[B286] Xu J. Bao T. Lee U. H. Kinnaird C. Carender W. Huang Y.. (2017). Configurable, wearable sensing and vibrotactile feedback system for real-time postural balance and gait training: proof-of-concept. J. Neuroeng. Rehabil. 14:102. 10.1186/s12984-017-0313-3 29020959 PMC5637356

[B287] Yang T. H. Kwon H. J. Lee S. S. An J. Koo J. H. Kim S. Y.. (2010). Development of a miniature tunable stiffness display using MR fluids for haptic application. Sensors Actuators A Phys. 163, 180–190. 10.1016/j.sna.2010.07.004

[B288] Yasuda K. Kaibuki N. Harashima H. Iwata H. (2017). The effect of a haptic biofeedback system on postural control in patients with stroke: an experimental pilot study. Somatosensory Motor Res. 34, 65–71. 10.1080/08990220.2017.1292236 28372470

[B289] Yekutiel M. Guttman E. (1993). A controlled trial of the retraining of the sensory function of the hand in stroke patients. J. Neurol. Neurosurg. Psychiatr. 56, 241–244. 8459238 10.1136/jnnp.56.3.241 PMC1014854

[B290] Yem V. Kajimoto H. (2017). Wearable tactile device using mechanical and electrical stimulation for fingertip interaction with virtual world, in IEEE Virtual Reality (VR), 99–104. 10.1109/VR.2017.7892236

[B291] Yem V. Otsuki M. Kuzuoka H. (2015). Development of a wearable out-covering haptic display using ball effector for hand motion guidance. Lecture Notes Electric. Eng. 277, 85–89. 10.1007/978-4-431-55690-9

[B292] Zandvliet S. B. Kwakkel G. Nijland R. H. M. van Wegen Erwin E. H. Meskers C. G. M. (2020). Is recovery of somatosensory impairment conditional for upper-limb motor recovery early after stroke? Neurorehabil. Neural Repair 34, 403–416. 10.1177/1545968320907075 32391744 PMC7222963

